# The Effectiveness of School‐Based Physical Activity Promotion on Mental Health Among Children and Adolescents: A Systematic Review

**DOI:** 10.1111/sms.70150

**Published:** 2025-10-19

**Authors:** Tia Viskari, Terhi Koivumäki, Kaija Appelqvist‐Schmidlechner, Timo Ståhl, Alberto Ruiz‐Ariza, Sari Fröjd

**Affiliations:** ^1^ Unit of Health Sciences, Faculty of Social Sciences Tampere University Tampere Finland; ^2^ Department of Public Health and Welfare Finnish Institute for Health and Welfare Helsinki Finland; ^3^ Faculty of Humanities and Educational Sciences University of Jaén Jaén Spain

**Keywords:** adolescents, children, mental health, physical activity, school, systematic review

## Abstract

Increasing evidence highlights the importance of schools in promoting both physical activity (PA) and mental health. However, previous findings on the effects of school‐based PA on the mental health of children and adolescents are mostly based on short‐term interventions. A synthesis of evidence is needed on the mental health effects of long‐term PA promotion, which is more sustainably integrated into the school structures. In addition, a more comprehensive examination of mental health, considering internalizing symptoms, externalizing symptoms, mental well‐being, and social well‐being, is needed. The aim of this systematic review was to investigate the effects of long‐term school‐based PA promotion on the mental health of 7–16‐year‐old children and adolescents. A literature search was conducted in six electronic databases. A total of 8795 unique articles were found, of which 38 articles describing 34 studies met the inclusion criteria. Regarding internalizing symptoms, externalizing symptoms, and mental well‐being, mixed results were found. However, the review showed a positive effect on social well‐being. Regarding the type of PA promotion, environmental modifications and PA promotion implemented by an external actor outside the school staff were found to be effective on mental health. PA promotion implemented by school staff without a specific protocol had no effect, while PA promotion implemented by school staff following a predefined protocol showed mixed results. Based on the results, it is recommended that schools invest in promoting PA to enhance the social well‐being of children and adolescents.

**Trial Registration:** PROSPERO registration number: CRD42022355274

## 1 Introduction

The health of children and adolescents is increasingly threatened by declining levels of physical activity (PA) and rising mental health problems. Over the past decades, mental health problems among children and adolescents have been reported to increase [1, 2, 3, 4, 5, 6], while the levels of PA have simultaneously declined [7]. The mental health benefits of PA among children and adolescents are already well established [8, 9, 10, 11, 12]. From a health promotion perspective, schools are considered an ideal setting for fostering PA, health, and well‐being among children and adolescents [13, 14]. There is also some evidence supporting the positive effects of school‐based PA on mental health [15, 16, 17, 18]. However, existing reviews offer inconsistent findings and lack a comprehensive view of how school‐based PA affects mental health. This systematic review aims to address this gap by synthesizing current evidence on the effectiveness of school‐based PA promotion on mental health.

The prevalence of mental health disorders is 8% in children aged 5–9 years and 14% in adolescents aged 10–19 years [19]. However, mental health is not only about problems but exists on a complex continuum encompassing mental health problems and mental well‐being [19]. While mental health problems refer to negative symptoms, mental well‐being refers to the positive aspects of mental health, and it is also referred to as positive mental health [20].

Symptoms of mental health problems in children and adolescents can be conceptualized as internalizing symptoms—comprising behaviors that are directed inward or are overcontrolled, such as depressed mood, anxiety, and emotional problems—and externalizing symptoms—referring to problematic behaviors such as impulsiveness, aggression, and hyperactivity [21]. In turn, mental well‐being is defined as a resource, encompassing subjective well‐being and functioning well in life. Mental well‐being consists of three dimensions: hedonic, eudaimonic, and social well‐being. The hedonic dimension refers to the individual's feelings towards his/her life, such as positive affects and satisfaction in life, while the eudaimonic dimension refers to functioning in life, such as being autonomous and competent. Furthermore, social well‐being refers to functioning well with other people [22, 23, 24]. Since the hedonic and eudaimonic dimensions of well‐being partially overlap [25, 26], and due to the wide range of measures used in studies making it difficult to distinguish them, this article will address the hedonic and eudaimonic dimensions together under the term ‘mental well‐being’. The social dimension of mental well‐being will be referred to as ‘social well‐being’.

As the onset of the first mental disorder occurs before the age of 14 in 35%, the age of 18 in 48%, and the age of 25 in 63% of individuals, it is essential to find ways to prevent mental health problems among children and adolescents [27]. Promoting mental well‐being is essential as well, as increases in the level of mental well‐being are associated with a decreased risk of future mental problems, and a reduction in mental well‐being is associated with an increased risk of future mental problems over a 10‐year period [28]. Mental health promotion consists of strengthening the ability to deal with thoughts and feelings, the management of life and emotional resilience, and the ability to deal with the social world [29]. The emphasis on promoting mental health among children and adolescents lies in social and emotional learning, including components such as self‐awareness, resilience, emotional regulation, problem‐solving, stress management, and interpersonal skills [30, 31].

One possibility for promoting the mental health of children and adolescents is through PA. PA is defined as any bodily movement produced by skeletal muscles that results in energy expenditure and encompasses all types of activity, exercise, and sports [32]. Children and adolescents aged 5–17 years are recommended to engage in at least 60 min of moderate‐to‐vigorous intensity physical activity (MVPA) daily. In addition, a regular muscle‐strengthening activity and reducing sedentary behaviors are recommended [33]. The physical inactivity of children and adolescents has been identified as a serious public health concern, as approximately 70% of the global population aged 5–17 does not meet the recommended amount of MVPA [34]. The volume of PA declines throughout childhood and adolescence, from the age of 7 years [35]. Globally, up to 81% of adolescents aged 11–17 years are insufficiently physically active [36].

The majority of children and adolescents can be reached in schools, as 91% of children and 86% of adolescents attend school globally [37], spending approximately half of their waking hours at school [38]. School‐based strategies, including PA promotion during the school day as well as before and after school, have shown to be important for PA engagement [39]. As school‐based programs are recommended for promoting mental health as well [19], research evidence on the association between school‐based PA promotion and mental health is needed.

Previous meta‐analysis investigating the effects of school‐related PA interventions on mental health in children and adolescents has reported that school‐based PA promotion reduced anxiety, while no effect on other internalizing symptoms was found [15]. In a recent review, the association between PA interventions in school and community settings and the mental ill‐being of children remained unclear [16]. Regarding mental well‐being, two reviews have found school‐based PA promotion effective [15, 16], while one reported no effect [17]. Furthermore, Schüller and Demetriou [18] found school‐based PA promotion effective in promoting social competence among children and adolescents. Despite the identified need for long‐term mental health promotion [40], the evidence provided by previous reviews is primarily based on short‐term interventions, as on average, 50% of the interventions included in these reviews last a maximum of 3 months, 70% a maximum of 6 months, and only 20% at least 9 months. In addition, a synthesis of research evidence on the association between school‐based PA promotion and externalizing symptoms is currently lacking. Thus, there is a need for a systematic review focusing on the effectiveness of long‐term school‐based interventions on the mental health of children and adolescents, considering the various aspects of mental health comprehensively. Furthermore, since previous reviews have identified challenges in aligning PA promotion with the curriculum, as well as deficiencies in time allocation and teachers' ability to implement PA promotion [41], a synthesis of evidence is needed to determine the most effective types of PA promotion in schools.

The aim of this systematic review is to investigate the effects of school‐based PA promotion on mental health among children and adolescents. Furthermore, this review seeks to identify the most effective forms of school‐based PA promotion for supporting mental health. The age group of 7–16 years was chosen for this study, as the majority of children and adolescents worldwide attend a similar type of school system during these years. The various aspects of mental health are thoroughly addressed, including internalizing and externalizing symptoms, as well as mental and social well‐being. Mental health, as a phenomenon, is best understood through the complementary use of both quantitative and qualitative data [42]. In particular, qualitative data are considered especially valuable in the context of children and adolescents [43]. To gain a multidimensional and comprehensive understanding of the topic, quantitative, qualitative, and mixed‐method primary studies are included. As long‐term mental health promotion among children and adolescents has been found to be more effective than short interventions [44, 45, 46], and the most successful school‐based health promotion interventions have been shown to have a duration of one school year [47], this review focuses on interventions lasting at least 9 months, which is the average length of a school year globally.

## 2 Methods

### 2.1 Protocol and Registration

This systematic review adheres to the Preferred Reporting Items for Systematic Reviews and Meta‐Analyses (PRISMA) guidelines [48]. However, as items 12 and 15 in the PRISMA guidelines are not fully applicable to a mixed‐methods review, they were implemented to the extent applicable. The review protocol was registered with PROSPERO (CRD42022355274). All the amendments made to the protocol have been reported in Supporting Information S1.

### 2.2 Search Strategy

The literature search was conducted on June 27, 2022, using six databases: Applied Social Sciences Index and Abstracts (ProQuest), ERIC (ProQuest), Medline (Ovid), PsycInfo (Ovid), SPORTDiscus (EBSCO), and Web of Science Core Collection. The search strategy included terms describing the population, PA promotion, study setting, and outcomes (see Table 1). The search was limited to peer‐reviewed articles published in English between January 1, 2012 and June 27, 2022. The search was repeated on March 28, 2024 for articles published between June 27, 2022 and March 28, 2024. Reference lists of included articles as well as previously published meta‐analyses and systematic reviews regarding the topic were screened for additional articles.

**TABLE 1:** Categories and search terms for the literature search.

Category	Search terms
Population	child* OR adolescent* OR teenage* OR young OR youth OR pupil* OR student*
Physical activity promotion	‘physical activity’ OR ‘physical training’ OR ‘physical education’ OR ‘physical fitness’ OR ‘motor activity’ OR sport* OR exercise* OR ‘physical inactivity’ OR sedentary
Setting	school*
Outcome	mental OR psychological OR psychosocial OR psychology OR well‐being OR wellbeing OR ‘quality of life’ OR emotion* OR mood OR self‐esteem OR self‐concept OR self‐worth OR self‐confidence OR self‐efficacy OR self‐perception OR self‐image OR resilience OR happiness OR ‘positive youth development’ OR coping OR ‘prosocial behavio*’ OR depress* OR anxiety OR ‘behavio* problem*’ OR ‘problem behavio*’ OR ‘social problem*’ OR aggression

### 2.3 Eligibility Criteria

All peer‐reviewed publications since January 2012 in the English language were eligible for inclusion. Since the results are intended to be applicable to current school systems, the search was limited to the past 10 years. Quantitative, qualitative, and mixed method primary studies were included regardless of the study design. However, case studies that involved fewer than five participants, conference/symposium abstracts and proceedings, theses, and study protocols were excluded.

Studies were included if they met the following inclusion criteria: (a) the participants' mean age ranged between 7 and 16 years; (b) the study includes school‐related PA promotion with a duration of at least 9 months aiming to increase PA (e.g., interventions, programs, curriculums, policies, environmental modifications, facilities, teaching practices). Activities operated before, during, and after school hours were included if they were clearly integrated into the school day or part of school activities. Actions operated by other stakeholders (e.g., sports clubs, parents, universities) were included if they were implemented in collaboration with the school. Studies including PA promotion as the only action as well as studies including PA with other health‐promoting actions (e.g., health education, nutritional actions) were included; and (c) the study reports mental health outcomes related to internalizing symptoms (e.g., depression, anxiety, emotional problems), externalizing symptoms (e.g., problem behavior, aggression, hyperactivity), mental well‐being (e.g., happiness, quality of life [QoL], self‐esteem, self‐efficacy), or social well‐being (e.g., prosocial behavior, relations with peers).

Studies were excluded based on the following criteria: (a) the study focuses purely on populations with specific mental disorders or physical conditions (e.g., overweight or obesity, physical disabilities, mental disorders, clinical populations); (b) actions comparing two or more different types of PA, but not increasing PA (e.g., comparing different pedagogies in physical education [PE] classes); or (c) mental health outcomes in the context of health behavior (e.g., physical self‐worth, eating self‐efficacy).

### 2.4 Study Selection

One reviewer (TV) conducted the searches and exported the references into Covidence. After duplicates were removed using Covidence, the titles and abstracts were initially screened against the inclusion criteria by two independent reviewers (TV and TK/SF/KAS/TS). The full texts of the remaining articles were independently screened by TV and TK. Any disagreements were resolved through discussion with a third independent reviewer (SF/KAS/TS).

### 2.5 Data Extraction

Detailed extraction of the relevant information was conducted by TV using a predefined data extraction form. A data extraction table was constructed in Microsoft Excel. Extracted information included the author(s), year of publication, country, study design, population characteristics (sample size, age, sex distribution, socioeconomic characteristics), details of the PA promotion (content, duration, deliverer), type of control or comparison group, outcome measures, and key findings related to mental health.

### 2.6 Quality Assessment

Study quality regarding the mental health outcomes was assessed using the mixed methods appraisal tool (MMAT) [49] which has been shown to be a valid and reliable tool for appraising quantitative, qualitative, and mixed methods research [50, 51]. The MMAT tool consists of two screening questions and 25 items for appraising the methodological quality of the studies in five different categories: (1) qualitative studies, (2) quantitative randomized controlled trials, (3) quantitative non‐randomized studies, (4) quantitative descriptive studies, and (5) mixed methods studies. Due to the nature of PA interventions and mental health variables, blinding would have been impossible in the studies of this review. As a result, the question regarding blinding in the randomized controlled trials' criteria was left unassessed. TV conducted the quality assessment based on the quantitative criteria, TK on the qualitative criteria, and both conducted the quality assessment based on the mixed method criteria.

### 2.7 Synthesis of Results

Schools as research environments present unique challenges for conducting tightly controlled randomized studies. Consequently, a variety of study designs are commonly employed in school‐based research. In order to gain as comprehensive an overview as possible of the current body of evidence, we chose to conduct a mixed method review that encompasses a wide range of study designs and outcomes. However, due to this methodological diversity, conducting a meta‐analysis was not feasible. Instead, a narrative synthesis was performed, which is an appropriate method when a review integrates data from diverse study designs [52]. Since many of the included studies were based on multi‐component programs that combined PA promotion with other elements, it is challenging to distinguish the specific effect of PA promotion on the outcomes. Therefore, a sensitivity analysis including only studies with single‐component programs focused solely on PA promotion was conducted.

### 2.8 Quality of Evidence

Due to the lack of established tools for assessing the certainty of evidence in integrated quantitative and qualitative findings [53, 54], certainty of evidence is not assessed in this mixed methods review. However, when interpreting the results of this systematic review, it is important to consider certain aspects related to the design, content, and quality of the included studies. The quality of the evidence was evaluated using the following criteria, adapted from Bray et al. [55]: (1) Does the evidence draw from both quantitative and qualitative data? (2) Is the evidence supported across different study designs? (3) Is the evidence based solely on children's self‐reported data, or does it also include assessments by adults (e.g., parents or teachers)? (4) Do the included studies cover the full age range (7–16 years) of the review? and (5) What is the quality of the studies on which the evidence is based?

## 3 Results

### 3.1 Study Selection

**FIGURE 1:**
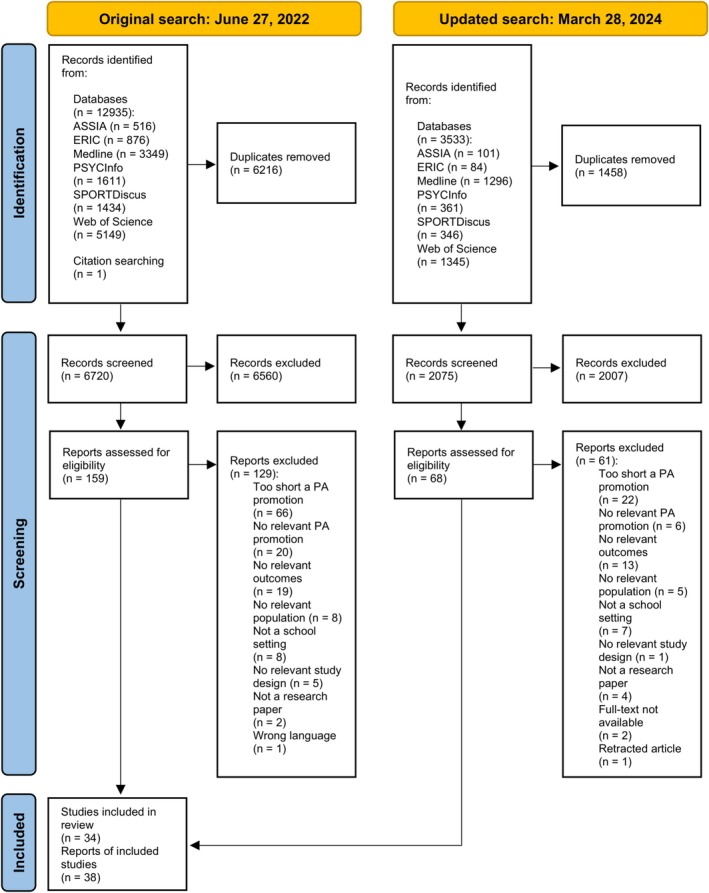
PRISMA flow diagram.

The original search resulted in 12 935 hits and following the removal of duplicates, 6719 unique articles remained. The screening of titles and abstracts identified 159 relevant articles for full‐text review. Finally, the full‐text review resulted in 30 eligible articles, whereas one article was found through the hand search. As a result of the original search, 31 eligible articles reporting 27 studies were found. The most common reasons for excluding the full‐text articles were too short a PA promotion, the absence of relevant PA promotion, and the absence of relevant outcomes. The updated search on March 28, 2024, resulted in 3533 hits, and following the removal of duplicates, 2075 unique articles remained. The screening of titles and abstracts identified 68 relevant articles for full‐text review. Finally, the full‐text review resulted in seven eligible articles. Following both searches, a total of 38 articles reporting 34 studies were included in the systematic review. A summary of the study selection process is presented in Figure 1.

Two articles examined the Whole of Systems Trial of Prevention Strategies for Childhood Obesity (WHO STOPS) study [56, 57], three articles focused on the Move for Well‐being in School (MWS) program [58, 59, 60], and two articles addressed the Girls Active program [61, 62]. In the synthesis of results, each of these was counted as a single study. Citations refer to one or more articles depending on the outcome variables reported.

### 3.2 Quality Assessment

The results of the quality assessment are detailed in Table 2. Sixteen studies were classified as quantitative RCTs. Among them, the most common quality concerns were inadequate reporting of the randomization process and adherence to the intervention. Thirteen studies were classified as quantitative non‐randomized studies, with the main quality concern being the participants' representativeness of the target population. In five of the qualitative studies, there were no quality concerns. One qualitative study was of low quality and did not pass the screening questions in addition to other quality concerns. Finally, in two of the mixed methods studies, quality concerns were found in the quantitative part of the study, while one study had minor concerns in the qualitative section.

**TABLE 2:** Quality assessment based on the mixed methods appraisal tool.

Authors (year)	Screening		Qualitative studies					Quantitative RCTs					Quantitative non‐randomized studies					Quantitative descriptive studies					Mixed methods studies				
	S1	S2	1	2	3	4	5	1	2	3	4	5	1	2	3	4	5	1	2	3	4	5	1	2	3	4	5
Allender et al. (2021)	✓	✓						?	?	✓	NA	✓															
Azevedo et al. (2014)	✓	✓											х	✓	✓	✓	✓										
Baker et al. (2017)	✓	✓	✓	✓	✓	✓	✓																				
Bandeira et al. (2021)	✓	✓						?	✓	✓	NA	?															
Barnes et al. (2021)	✓	✓						✓	✓	✓	NA	?															
Bunketorp Käll et al. (2015)	✓	✓											✓	✓	х	✓	✓										
Carter et al. (2017)	✓	✓											х	✓	✓	✓	?										
Christiansen et al. (2018)	✓	✓						✓	✓	✓	NA	✓															
Christiansen et al. (2022)	✓	✓						✓	✓	✓	NA	✓															
Diao et al. (2020)	✓	✓						?	✓	✓	NA	✓															
Elinder et al. (2012)	✓	✓											х	✓	?	?	х										
Grillich et al. (2016)	✓	✓						✓	✓	✓	NA	?															
Hall et al. (2022)	✓	✓						✓	✓	х	NA	✓															
Hamer et al. (2017)	х	?	?	?	✓	✓	✓																				
Harrington et al. (2018)	✓	✓						✓	✓	✓	NA	?															
Harrington et al. (2019)	✓	✓						✓	✓	✓	NA	?															
Holt et al. (2012)	✓	✓	✓	✓	✓	✓	✓																				
Jacobs et al. (2021)	✓	✓						?	✓	✓	NA	?															
Jägerbrink et al. (2022)	✓	✓	✓	✓	✓	✓	✓																				
Kliziene et al. (2023)	✓	✓											?	✓	?	х	?										
Kvalo & Natlandsmyr (2021)	✓	✓						✓	✓	✓	NA	✓															
Lazardis et al. (2023)	✓	✓	✓	✓	✓	✓	✓																				
Lubans et al. (2012)	✓	✓						?	✓	✓	NA	х															
Lubans et al. (2021)	✓	✓						✓	✓	х	NA	✓															
Malakellis et al. (2017)	✓	✓											х	✓	✓	✓	х										
Masini et al. (2023)	✓	✓											?	✓	✓	х	х										
Meyer et al. (2014)	✓	✓						✓	✓	х	NA	✓															
Mills et al. (2015)	✓	✓											х	х	?	✓	✓										
Nathan et al. (2013)	✓	✓	✓	✓	✓	✓	✓						х	✓	✓	?	х						✓	✓	✓	✓	х
Rocher et al. (2020)	✓	✓	✓	✓	✓	✓	?											✓	✓	✓	✓	✓	х	✓	✓	✓	х
Ryom et al. (2021)	✓	✓	✓	✓	✓	✓	✓						✓	✓	х	?	х						✓	✓	✓	✓	х
Schirmer et al. (2023)	✓	✓	✓	✓	✓	✓	✓																				
Smedegaard et al. (2017)	✓	✓						✓	✓	✓	NA	✓															
Tennfjord et al. (2023)	✓	✓											✓	✓	х	✓	?										
van Dijk‐Wesselius et al. (2018)	✓	✓											?	✓	✓	✓	✓										
Villarreal & Gonzalez (2016)	✓	✓											х	✓	?	✓	✓										
Woodgate & Sigurdson (2015)	✓	✓											х	✓	✓	х	х										
Zhou et al. (2023)	✓	✓											?	✓	✓	х	✓										

Abbreviations: ?, Can not tell; ✓, Yes; NA, not assessed; х, No.

### 3.3 Study Characteristics

The characteristics of the included studies are presented in Table 3. The 34 studies involved a total of 21 651 participants. The sample size varied from 26 to 2797. The age range of the target group was 6–21 years, but the mean age in all studies was between 7 and 16 years. The majority of the studies (*n* = 26) included both sexes, while two studies included only girls and one study included only boys. Socioeconomic status was reported to be low in nine studies, mixed in 10 studies, and high/moderate in four studies. Sex distribution was not reported in five studies and socioeconomic status in 11 studies.

**TABLE 3:** Characteristics of included studies.

Authors (year)	Country	Study design	Population	Physical activity promotion	Control/comparison group	Mental health outcome(s)	Key findings
Allender et al. (2021) Jacobs et al. (2021)	Australia	cRCT	Primary school children from 10 communities in South West Victoria Sample: *n*_IG_ = 970, *n*_CG_ = 820 Age: 6–13 years, M_age_ = 9.8 years Sex F/M (%): 48.3/51.7 SES: Mean Socio‐Economic Indexes For Areas (SEIFA) score: 978	Name: Whole of Systems Trial of Prevention Strategies for Childhood Obesity (WHO STOPS) Content: Community‐based intervention included drop‐off zones around schools to encourage children to walk the last 800 m, construction of a new footpath enabling improved active transport to school, walking school buses, running groups, running events, teaching bike safety, and actions to promote healthy eating Duration: 4 years	Waitlist control	Psychosocial HRQoL	Statistically significant between‐group effect on psychosocial HRQoL favoring IG in 4‐year follow‐up. No statistically significant between‐group effect on psychosocial HRQoL in 2‐year follow‐up
Azevedo et al. (2014)	UK	Quasi‐experimental study	Secondary school students from seven urban schools in the north‐east of England Sample: *n*_IG_ = 280, *n*_CG_ = 217 Age: 11–12 years, M_age_ = 11.2 years Sex F/M (%): 64.2/35.8 SES: Mean index of multiple deprivation rank: 9872. Mean free school meals eligibility (%): 35.1	Content: Dance mat systems provided to schools, 6 weeks' structured delivery of the dance mats into the curriculum, after that schools had the freedom to use the mats in a way they wanted Duration: Permanent action at intervention schools. Baseline data collection in September 2010, mats were introduced at schools in January 2011, follow‐up data collection in March 2012	Usual practice	Psychological well‐being	Statistically significant between‐group effect on psychological well‐being favoring IG
Baker et al. (2017)	USA	Qualitative study	Elementary school students from four rural schools in south‐east Missouri Sample: Interviews: *n* = 5 administrators, *n* = 4 teachers Age: Not reported Sex F/M (%): Not reported SES: Not reported	Name: Healthier Missouri Communities (Healthier MO) Content: Environmental changes: Installing asphalt walking tracks at four schools and the purchase of playground and PA equipment at one school. Policy changes: Implementing brain breaks in the classrooms Duration: Permanent action at intervention schools. Data collection in 1‐year follow‐up	None	Behavioral changes	Those interviewed reported that they saw fewer behavioral problems and increased student focus and engagement after implementing brain breaks
Bandeira et al. (2021)	Brazil	cRCT	Elementary school students from six schools in Florianopolis Sample: *n*_IG_ = 538, *n*_CG_ = 383 Age: 10–16 years Sex F/M (%): 51.5/48.5 SES: Mean SES score (from 0 to 15, higher values referring to greater family wealth): 4.9	Name: Movimente Program Content: The program includes three components: teacher training, active opportunities in the school environment (sports equipment kits), and health education materials. Duration: One school year (9 months)	Usual practice	Psychological well‐being	No statistically significant between‐group effect on pshychological well‐being
Barnes et al. (2021)	Australia	cRCT	Students from 12 Catholic primary schools in New South Wales Sample: *n*_PA_ = 283, *n*_NUTR_ = 163, *n*_COMB_ = 202, *n*_CG_ = 167 Age: Fourth to sixth graders Sex F/M (%): 51.8/48.2 SES: Socio‐Economic Indexes For Areas (SEIFA) classification, most disadvantaged (%): 69.1	PA group: Physically Active Children in Education (PACE) Content: Scheduling of 150 min of planned PA (physical education, sport, energisers, active lessons) across the school week Duration: 9 months Nutrition group: SWAP IT Content: Supporting parents to improve the nutritional quality of children's lunchboxes Duration: 5–6 months Combined group: Both interventions received concurrently	Waitlist control	Social functioning Emotional functioning	No statistically significant between‐group effect on social functioning or emotional functioning between the four groups, neither between the students receiving PA intervention nor the students not receiving PA intervention
Bunketorp Käll et al. (2015)	Sweden	Quasi‐experimental study	Students from two elementary schools in Mölndal Sample: *n*_IG_ = 182, *n*_CG_ = 167 Age: Preschoolers to sixth graders M_age_ = 10.0 years Sex F/M (%): 47.9/52.1 SES: Not reported	Name: School‐in‐Motion Content: In addition to two standard curricular PE lessons, students in intervention school had two mandatory PE lessons per week, 30–45 min each Duration: Permanent action at intervention school. Students had participated the program from their preschool class	Usual practice	Psychological well‐being Emotional and behavioral problems	0–3‐graders: No statistically significant between‐group effect on any mental health outcomes. No subgroup analysis reported 4–6‐graders: Statistically significant between‐group effect on conduct problems favoring IG. No other statistically significant between‐group effects on mental health outcomes Subgroup analysis: Statistically significant between‐group effect on hyperactivity favoring IG among girls. No other statistically significant between‐group effects among girls or boys
Carter et al. (2017)	USA	Quasi‐experimental study	Students from four low‐income, public elementary schools in an urban, Midwestern metropolis in USA Sample: *n*_IG_ = 78, *n*_CG_ = 33 Age: Third graders, M_age_ = 8.5 years Sex F/M (%): 56.0/44.0 SES: Schools were serving high numbers of low‐income, Latino, and Black youth	Name: The Urban Initiatives Work to Play Content: Three soccer practices per week, 60 min each, and additionally 20 min of nutrition, health, and character‐building discussions Duration: Intervention included 24 weeks of practice in total, throughout the school year. Data collection in 9‐month follow‐up	Waitlist control	Internalizing symptoms, Externalizing symptoms	Statistically significant between‐group effect on internalizing symptoms favoring IG. No statistically significant between‐group effect on externalizing symptoms
Christiansen et al. (2018) Christiansen et al. (2022) Smedegaard et al. (2017)	Denmark	cRCT	Students from 24 public schools in seven municipalities in Denmark Sample: *n*_IG_ = 1301, *n*_CG_ = 1496 Age: 10–13 years Sex F/M (%): 48.9/51.1 SES: Family social class (%): upper‐middle: 41.0, middle: 47.2, lower‐middle: 11.8	Name: The Move for Well‐being in School (MWS) Content: A multi‐component PA promotion program targeting four settings: (1) PE classes (half of PE classes taught according to the MWS lesson plans), (2) in‐class activities (5 min brain breaks twice a day), (3) recess activities (30 min three times a week), and (4) theme days (3 days focusing on well‐being and PA) Duration: 9 months	Usual practice	Social competence, Global self‐worth Psychological well‐being Well‐being promotion	No statistically significant between‐group effect on social competence or global self‐worth Subgroup analysis: Statistically significant between‐group effect on global self‐worth favoring IG among those students who did not participate in leisure sport Statistically significant between‐group effect on psychological well‐being favoring CG among those who were most active at baseline 75% of the educators reported that the intervention improved well‐being among their students
Diao et al. (2020)	China	cRCT	Students from two primary and two middle schools in ChongQing Sample: *n*_IG_ = 547, *n*_CG_ = 445 Age: 9–17 years, M_age_ = 11.4 years Sex F/M (%): 48.1/51.9 SES: Family economic status (%): poor: 12.3, medium: 61.4, good: 26.4	Content: A multi‐component family‐, individual‐, and school‐based intervention program involving three aspects: health education, diet, and PA. PA promotion at school included Sunshine Sports Activities through utilizing a big break between classes and promising physical exercise for 1 h per day Duration: 1 year	Usual practice	Psychological QoL Social QoL	Statistically significant between‐group effect on psychological QoL favoring IG. No statistically significant between‐group effect on social QoL
Elinder et al. (2012)	Sweden	Quasi‐experimental study	Students from 18 schools in Österåker Sample: *n*_IG_ = 482, *n*_CG_ = 331 Age: Second, fourth, and seventh graders Sex F/M (%): Not reported. SES: Schools were located in a middle‐class municipality.	Name: The Stockholm County Implementation Programme in school (SCIP‐school) Content: The health team from every intervention school made their own action plan to their school. The aim of the program was to improve eating habits, PA, and self‐esteem, and to promote a healthy body weight in children. Workshops, training, feedback, materials, etc., were provided to support health teams Duration: 2 years	Usual practice	Self‐esteem	No statistically significant between‐group effect on self‐esteem
Grillich et al. (2016)	Austria	cRCT	Students from 53 classes from 45 primary schools in Lower Austria Sample: *n*_IG_ = 432, *n*_CG_ = 493 Age: 8–9 years, M_age_ = 8.7 years Sex F/M (%): 50.9/49.1 SES: Socio‐economic level (%): low: 10.0, middle: 74.1, high: 55.1	Name: Classes in Motion Content: 20 h of training and two 8‐hour workshops aimed to develop teacher competency, create a healthy and positive learning environment, and improve the quality of PE classes. Training included topics related to PA, for example, active teaching Duration: 1.5 academic years	Waitlist control	Psychological well‐being Moods and emotions Sense of coherence	No statistically significant between‐group effect on psychological well‐being, moods and emotions, or sense of coherence
Hall et al. (2022)	Australia	cRCT	Students from 61 government and Catholic schools in the Hunter New England region Sample: *n*_IG_ = 1282, n_CG_ = 1203 Age: 7–9 years, M_age_ = 8 years Sex F/M (%): 51.9/48.1 SES: Socio‐Economic Indexes For Areas (SEIFA) disadvantage classification most/least disadvantaged (%): 56.7/43.3	Name: Physically Active Children in Education (PACE) Content: Supporting schools in impelementation of the PA policy, with a minimum on 150 min PA across the school week. Intervention includes assistance and training, educational visits, development of materials, etc. Duration: 1 year	Waitlist control	Psychosocial HRQoL	No statistically significant between‐group effect on psychosocial HRQoL
Hamer et al. (2017)	UK	Quasi‐experimental study	Students from seven primary schools in the inner city of London Sample: *n*_IG_ = 169, *n*_CG_ = 62. Interviews: *n* = 12 students, *n* = 2 teachers, *n* = 2 parents Age: M_age_ = 8 years Sex F/M (%): 45/55 SES: Schools were located in deprived areas	Name: Camden Active Spaces Content: Major playground constructions. Playground areas were designed to be conductive to PA via active play Duration: Permanent action at intervention schools. Data collection in 1‐year follow‐up	Usual practice	Self‐efficacy, Well‐being, Social interactions	New playgrounds had positive repercussions on perceived self‐efficacy, well‐being, and social interactions
Harrington et al. (2018) Harrington et al. (2019)	UK	cRCT	Female students from 20 government‐funded secondary schools in the Midlands Sample: *n*_IG_ = 867, *n*_CG_ = 885 Age: 11–14 years, M_age_ = 12.8 years Sex F/M (%): 100/0 SES: Mean index of multiple deprivation (IMD) decile score (from 1 to 10, where 1 is the least and 10 the most deprived): 5.8	Name: Girls Active Content: A multi‐component intervention involving teachers reviewing PA, sport, and PE provision, culture and practices in their school, training, creating action plans, and working with peer leaders to influence decision‐making and promote PA to their peers. Support from a hub school and the Youth Sport Trust organization is offered Duration: 14 months	Usual practice	Self‐esteem	14‐month follow‐up: No statistically significant between‐group effect on self‐esteem
Holt et al. (2012)	Canada	Qualitative study	Students from one inner‐city elementary/junior high school in a western Canadian city Sample: Interviews: *n* = 8 school staff members, *n* = 59 students Age: Fifth to ninth graders, M_age_ = 12.4 years Sex F/M (%): 47.5/52.5 SES: The school was located in a low‐income neighborhood	Content: PE and sport programs: PE lessons, intramural lunchtime sports, and school sport teams Duration: Permanent actions at intervention schools. Data collected during one school year. Intramural lunchtime sports were canceled after 7 months	None	Empathy, Social connections, Problem behavior	PE and sport programs offered opportunities to learn empathy and make social connections, but intramural lunchtime sports was associated with negative behaviors
Jägerbrink et al. (2022)	Sweden	Qualitative study	Students from two municipal schools in two cities Sample: Interviews: *n* = 44 students Age: 10–15 years Sex F/M (%): 50.0/50.0 SES: Not reported	Content: Mandatory extracurricular PA with an intention to reach a pulse rate of 70% Duration: Interviewed students had participated in activities for nearly two school years	None	Sense of happiness and/or wellbeing, Stress	Interviewed students reported enhanced sense of happiness and/or wellbeing, and to some extent, reduced stress
Kliziene et al. (2023)	Lithuania	Pre‐post study	Students from three schools representing the state education system Sample: *n* = 202 Age: 9–10 years Sex F/M (%): 49.5/50.5 SES: Not reported	Content: PE program: Three 45‐min lessons per week, including activities promoting health, fitness, skills, relaxation, focus and reflection. In addition, the program provided material and devices Duration: 9 months	None	Anxiety	Statistically significant difference between pre‐test and post‐test in personality anxiety scale and social anxiety scale for both boys and girls, and in somatic anxiety scale for boys, indicating that the PE program reduced anxiety
Kvalo and Natlandsmyr (2021)	Norway	cRCT	Students from nine schools in Stavanger Sample: *n*_IG_ = 227, *n*_CG_ = 231 Age: 10–11 years Sex F/M (%): Not reported SES: Not reported	Name: Active School Content: In addition to 135 min of curricular PA, intervention schools provided two 45‐min PA outdoor lessons, five 10‐min PA breaks, and five 10‐min PA homework tasks every week Duration: 11 months	Usual practice	Psychological well‐being	Statistically significant between‐group effect on psychological well‐being favoring IG
Lazaridis et al. (2023)	Greece	Qualitative study	Students from one secondary school in the city centre of Athens Sample: *n* = 75. Interviews: *n* = 12 students Age: 12–14 years Sex F/M (%): 50.0/50.0 SES: Students with mixed backgrounds	Content: Outdoor activities (games invented by students) in the schoolyard once a week after the school. In addition, students participated in three trips during the school year, including adventurous sports, such as hiking, rock climbing, and canyoning Duration: 2 years	None	Self‐confidence, Happiness Relations with peers	Interviewed students reported improved self‐confidence, happiness, and relationships with peers
Lubans et al. (2012)	Australia	cRCT	Female students from 12 state‐funded government secondary schools in low‐income communities in New South Wales Sample: *n*_IG_ = 178, *n*_CG_ = 179 Age: 12–14 years, M_age_ = 13.2 years Sex F/M (%): 100/0 SES: Socioeconomic position (%, from 1 to 10, where 1 is the lowest and 10 the highest): 1–2: 21.1, 3–4: 24.5, 5–6: 51.3, 7–8: 2.5, 9–10: 0.3	Name: Nutrition and Enjoyable Activity for Teen Girls (NEAT Girls) Content: A multi‐component program including teacher development, enhanced school sport sessions, interactive seminars, nutrition workshops, lunch‐time PA sessions, handbooks and pedometers for self‐monitoring, parent newsletters, text messaging for social support, and sports equipment packs Duration: 1 year	Usual practice	Global self‐esteem	No statistically significant between‐group effect on global self‐esteem
Lubans et al. (2021)	Australia	cRCT	Students from 20 government secondary schools in New South Wales Sample: *n*_IG_ = 337, *n*_CG_ = 333 Age: 11th graders, M_age_ = 16.0 years Sex F/M (%): 44.6/55.4 SES: Socioeconomic status (%): low: 19.4, medium: 51.1, high: 29.5	Name: Burn 2 Learn (B2L) Content: A high intensity interval training (HIIT) intervention including training, resources, and support for teachers; an information seminar, a smartphone application, and heart rate monitors for students; and newsletters for parents Duration: 1 year	Usual practice	Perceived stress Psychological difficulties Mental well‐being	No statistically significant between‐group effect on any of the mental health outcomes
Malakellis et al. (2017)	Australia	Quasi‐experimental study	Secondary students from six government schools in Canberra Sample: *n*_IG_ = 628, *n*_CG_ = 252 Age: 12–16 years, M_age_ = 13.1 years Sex F/M (%): 53.3/46.7 SES: Schools were located in an area that is ranked as most relatively advantaged and least relative disadvantaged compared to other states and territories	Name: Australian Capital Territory “It's your move!” (ACT‐IYM) Content: A multi‐component systems intervention including school food policy and PA promotion (e.g., increasing the time spent on PA and active transport to and from school). Each three schools had unique objectives Duration: 3 years	While intervention schools received $50 000 covering the costs, the control schools received $5000	Depressive symptoms	Statistically significant between‐group effect on depressive symptoms favoring school C (whose objective was to increase mental well‐being through the promotion of healthy eating and PA) compared to other two intervention schools combined. Depressive symptoms were not analyzed between IG and CG
Masini et al. (2023)	Italy	Quasi‐experimental study	Students from one primary school in Imola Sample: *n*_IG_ = 83, *n*_CG_ = 50 Age: M_age_ = 7.61 years Sex F/M (%): 47.4/52.6 SES: The majority of the participants' parents had a medium or high level of education	Name: Imola Active Breaks (I‐MOVE) Content: Three 10 min active breaks per day. Each active break consisted of 2 min warm‐up, 5 min high intensity interval training (HIIT), and 3 min cool‐down Duration: 1.5 years	Usual practice	Psychosocial HRQoL	No statistically significant between‐group effect on psychosocial HRQoL
Meyer et al. (2014)	Switzerland	cRCT	Students from 15 schools in Switzerland Sample: *n*_IG_ = 297, *n*_CG_ = 205 Age: First and fifth graders, M_age_ = 8.5 years Sex F/M (%): 54.7/45.3 SES: Not reported	Name: Kinder‐ und Jugendsportstudie (KISS) Content: A multi‐component program including two additional PE lessons per week, 3–5 PA breaks, 2–5 min each, during lessons every day, and daily 10 min PA homework Duration: 9 months	Usual practice	Psychological QoL	Three‐year follow‐up: No statistically significant between‐group effect on psychological QoL
Mills et al. (2015)	UK	Quasi‐experimental study	Students from 12 schools in the Midlands and north‐west England Sample: *n*_IG_ = 249, *n*_CG_ = 146 Age: M_age_ = 11.5 years Sex F/M (%): 44.8/55.2 SES: Schools included in the top 20% most deprived nationally, and had a high percentage of pupils eligible for free school meals	Name: Commando Joe's Content: A military‐ethos PA intervention including weekly sessions (team building, problem solving, and fitness activities) and extracurricular activities (late‐attendace monitoring, math and English booster classes, mentoring, and PA sessions) Duration: 1 year	Allocation and practices of control group are not reported	Positive social behavior, Problem behavior	1‐year follow‐up: Statistically significant between‐group effect on both positive social behavior subscales favoring IG Statistically significant between‐group effect on self‐injury/stereotypic behaviors favoring IG, and on conduct problems favoring CG. No statistically significant between‐group effect on the other subscales of problem behavior
Nathan et al. (2013)	Australia	Quasi‐experimental study	Students who had arrived in Australia as refugee or humanitarian entrants, from four preparitory schools for newly arrived secondary age immigrants and their host high schools in western Sydney Sample: *n*_IG_ = 63, *n*_CG_ = 79 Age: M_age_ = 14.7 years Sex F/M (%): 22.5/77.5 SES: Vulnerable population with trauma and persecution experiences	Name: Football United Content: A multi‐level football intervention including regular Saturday and after‐school football training, other football activities, capacity and linkage building, and creating awareness Duration: Intervention schools had participated in the program prior to and during the study. 79% of participants had pariticipated in the program already in previous year	Waitlist control	Emotional symptoms, Hyperactivity, Peer problems, Prosocial behavior, Resilience	Survey: No statistically significant between‐group effect on any mental health outcomes Subgroup analysis: Significant between‐group effect on peer problems and prosocial behavior favoring IG among boys. No significant between‐group effect on any other mental health outcomes among boys. No subgroup analysis done among girls Interviews: The quantitative results were well supported by the qualitative data. Interviewed students in IG reported making new friends and overcoming peer problems, while students in CG reported difficulties connecting with friends after school
Rocher et al. (2020)	Portugal	Case study	Students from elementary, middle, and high schools in Viana do Castelo, who had been enrolled from the beginning of the project Sample: *n* = 595, Interviews: *n* = 14 students, *n* = 16 parents, *n* = 5 teachers Age: 11–21 years, M_age_ = 14.0 years Sex F/M (%): 55.5/44.2 SES: Not reported	Name: School Nautical Activity project Content: Nautical acitivities (surfing, rowing, sailing, and canoeing) program in blue spaces at least 45 min once a week Duration: A four‐year program, study participants has been participating in the program for an average of 2.4 years	None	Mental health and well‐being Social behavior, Sense of community	The program was found to be beneficial to the mental health and well‐being, social behavior, and sense of community of the participants
Ryom et al. (2021)	Denmark	Quasi‐experimental study	Male students from two public schools in Copenhagen, with the majority of students having a migrant background Sample: *n*_IG_ = 89, *n*_CG_ = 40, Interviews: *n* = 12 students Age: 12–16 years, M_age_ = 14.2 years Sex F/M (%): 0/100 SES: Schools were located in a low socioeconomic status area	Content: A group coaching/mentoring and football intervention including 20–25 coaching sessions (45 min each, during school hours) per year addressing different themes (society, school, family, life history, religion, and friendship), and three weekly football training classes, 1–1.5 h each, before and after school hours Duration: 2 years	Practices of control group are not reported	General self‐concept, Relations to peers Social relations	Questionnaire: Statistically significant between‐group effect on general self‐concept favoring IG. No statistically significant between‐group effect on relations to peers or social relations Interviews: Participants from the IG reported stronger and more confident self and stronger and more supportive social environment
Schirmer et al. (2023)	Australia	Qualitative study	Students from four primary schools across the Mid North Coast in New South Wales Sample: Interviews: *n* = 26 families (students and their parents/carers), *n* = 4 teachers, *n* = 2 principals Age: Not reported Sex F/M (%): Not reported SES: Not reported	Name: Kilometre Club (KM Club) Content: Running, walking, or jogging on an outside course, 15–30 min from 2 to 5 days a week Duration: 5–10 months.	None	Social connection, Anxiety	Interviewed students and parents/carers reported improved social connections. Interviewed parents/carers and teachers reported reduced anxiety levels among students participating in the KM Club
Tennfjord et al. (2023)	Norway	Quasi‐experimental study	Students from seven elementary schools in Horten (intervention schools) and two schools in Viken County (control schools) Sample: *n* = 1221 Age: 11–12 years Sex F/M (%): Not reported for full sample SES: Not reported for full sample	Name: The Health Oriented Pedagogical Project (HOPP) Content: An additional 45 min of PA each day, replacing ordinary desk learning with physical tasks Duration: 4 years, but only 1 year of follow‐up data was collected from each student	Usual practice	Internalizing difficulties, Externalizing difficulties, Total difficulties	Statistically significant between‐group effect on internalizing difficulties favoring CG in year 2017 follow‐up, on externalizing difficulties favoring CG in the 2019 follow‐up, and on total difficulties favoring the CG in the 2016, 2017, and 2019 follow‐ups
van Dijk‐Wesselius et al. (2018)	The Netherlands	Quasi‐experimental study	Students from nine elementary schools in urbanized areas of the Netherlands Sample: *n*_IG_ = 351, *n*_CG_ = 355 Age: 7–11 years, M_age_ = 8.6 years Sex F/M (%): 50.3/49.7 SES: Not reported	Content: Schools greened their schoolyards between pre‐measurement and first follow‐up. Green areas covered mostly features such as grassy hills, bushes, trees, tunnels made of tree branches, loose tree branches, and garden‐like parts Duration: Permanent action at intervention schools. Data collection in 1‐year and 2‐year follow‐ups	Usual practice	Prosocial orientation Peer problems, Prosocial behavior Social support Emotional functioning	Statistically significant between‐group effect on peer problems and social support favoring IG in 1‐year follow‐up, and on social support in 2‐year follow‐up No statistically significant between‐group effect on prosocial orientation, prosocial behavior, or emotional functioning in either follow‐up, or on peer problems in the 2‐year follow‐up
Villarreal and Gonzalez (2016)	USA	Cross‐sectional study	Hispanic middle school students from Texas Sample: *n* = 186 Age: Seventh graders Sex F/M (%): 47.3/52.7. SES: 79.6% of the participants were economically disadvantaged	Content: Participants were asked whether they had participated (after school or during weekends) in school‐based extracurricular sports or nonsports (fine arts or performance clubs, academic clubs, government, service clubs) activities Duration: Extracurricular activity was a permanent action at schools	Those who had participated in nonsports activities	Prosocial behavior Prosocial peer affiliation	Participation in sports activities or nonsport activities was not associated with prosocial behavior. Participation in sports activities was associated with prosocial peer affiliation, while participation in nonsport activities was not
Woodgate and Sigurdson (2015)	Canada	Pre‐post study	Students from one class in one urban middle school in Canada Sample: *n* = 26 Age: 12–13 years Sex F/M (%): 46.2/53.8 SES: Youth enrolled in the school district had diverse cultural and socioeconomic backgrounds	Name: Health Experts and Research Team (HEART) Content: Education, empowerment, and support to build youth's knowledge and skills for identifying, initiating, leading, and monitoring cardiovascular health promotion activities in four areas: smoking, physical inactivity, nutrition, and obesity Duration: Two school years (22 months)	None	Positive youth development	There were no statistically significant changes in any of the five positive youth development (PYD.2) subscales
Zhou et al. (2023)	China	Pre‐post study	Left‐behind children students from six primary schools in rural areas of Hunan province Sample: *n* = 367 Age: 9–13 years, M_age_ = 10.5 years Sex F/M (%): 47.5/52.5 SES: Not reported	Content: Latino dance lessons, 1.5 h each, twice per week, 144 h in total Duration: 1.5 years	None	General self‐efficacy Self‐esteem	Statistically significant difference between pre‐test and post‐test in general self‐efficacy and self‐esteem, indicating that the Latino dance intervention improved general self‐efficacy and self‐esteem

Abbreviations: CG, control group; COMB, combined; cRCT, cluster randomized controlled trial; F, female; HRQoL, health‐related quality of life; IG, intervention group; M, male; M_age_, mean age; *n*, sample size; NUTR, nutrition; PA, physical activity; PE, physical education; QoL, quality of life; SES, socioeconomic status.

The included studies were from 16 different countries. Eight studies were conducted in Australia; four in the United Kingdom; three in Sweden and the USA; two in Canada, China, Denmark, and Norway; and one study in Austria, Brazil, Greece, Italy, Lithuania, the Netherlands, Portugal, and Switzerland. The study designs included 12 cluster‐randomized controlled trials, 12 quasi‐experimental studies, three pre‐post studies, one cross‐sectional study, one case study, and five qualitative designs.

There was a large variation in type, content, mode of delivery, and duration of the PA promotion. Thirteen studies contained a multi‐component program including PA promotion with other component(s), such as health education, healthy eating, mentoring, and environmental modifications. Nineteen studies contained a program solely focusing on PA promotion. Two studies addressed environmental changes exclusively. In this review, PA promotion was categorized into four different types as ‘PA promotion including permanent environmental modifications’ (*n* = 5), ‘PA promotion delivered by an external actor outside the school staff’ (*n* = 7), ‘PA promotion delivered by school staff following a specific, predefined protocol’ (*n* = 10), and ‘PA promotion delivered by school staff without any specific protocol’ (*n* = 9). Three studies could not be placed in the categories mentioned above, since one study did not report the deliverer of the program [63] and in two studies the programs were partially delivered by school staff and partially by external actors [64, 65].

Control and comparison groups received usual practice (*n* = 15), other (non‐PA) activities (*n* = 2), or were a waitlist control group (*n* = 6). Usual practice refers to the control group following existing school routines without any modifications to daily practices. Accordingly, the control group was not exposed to PA promotion or any other form of intervention. Two studies did not report the practices of the control group, and nine studies had no control or comparison group. Only in seven of the studies was the aim of the PA actions to promote mental health. In the other 27 studies, mental health was measured as a secondary outcome. In these studies, PA promotion was targeted towards increasing PA (*n* = 7), promoting overall health (*n* = 6), promoting physical health (*n* = 5), preventing overweight (*n* = 5), promoting educational outcomes (*n* = 2), or increasing social capital (*n* = 2).

### 3.4 Synthesis of Effects on Mental Health

For quantitative articles, only statistically significant results were considered in this synthesis of findings. Of the 34 studies including a school‐based PA promotion, 22 (13 quantitative, seven qualitative, and two mixed methods) reported positive effects on at least one mental health outcome, while 11 quantitative studies found no effect on mental health outcomes. Three studies (two quantitative and one qualitative) found both positive and negative effects, and one quantitative study reported no effect at some time points and negative effects at others.

#### 3.4.1 Internalizing and Externalizing Symptoms

Internalizing symptoms were assessed in 12 studies (nine quantitative, two qualitative, and one mixed methods) using eight different instruments and interviews. Internalizing symptoms were examined from various perspectives, including depressive symptoms, anxiety, emotional symptoms, emotional functioning, moods and emotions, perceived stress, and overall internalizing symptoms. Six studies (five quantitative and one mixed methods) reported no effect, while five studies (three quantitative and two qualitative) found a positive effect on at least one outcome representing internalizing symptoms. One quantitative study reported mixed results. The numerical data from quantitative studies concerning internalizing symptoms is provided in Table S1 of Supporting Information S2. Five studies reported no effect on outcomes related to moods and emotions [66, 67, 68, 69, 70]. Jägerbrink et al. [63] reported a positive effect on stress, while Lubans et al. [71] found no effect. Carter et al. [72] found a positive effect on internalizing symptoms, while Lubans et al. [71] reported no effect, and Tennfjord et al. [73] reported no effect at three time points and a negative effect at one time point. Two studies reported a positive effect on anxiety [65, 74]. One study [75] reported a positive effect on depressive symptoms at one intervention school with the objective of increasing mental well‐being through the promotion of healthy eating and PA, compared to the other two intervention schools with the objectives of increasing PA at school and increasing active transport to and from school. However, depressive symptoms were not analyzed comparing the intervention and control groups.

Externalizing symptoms were assessed in seven studies (four quantitative, two qualitative, and one mixed methods) using four different instruments, interviews, and observations. The included studies examined various perspectives of externalizing symptoms, including behavioral problems in general, conduct problems, hyperactivity, and overall externalizing symptoms. Two studies (one quantitative and one qualitative) found positive effects on at least one outcome representing externalizing symptoms, while two studies (one quantitative and one mixed methods) reported no effect. One qualitative study found a negative effect, and two quantitative studies reported mixed results. The numerical data from quantitative studies concerning externalizing symptoms is provided in Table S2 of Supporting Information S2. Baker et al. [76] reported decreased behavioral problems, while Holt et al. [77] found that intramural sports, which were one part of the PA program, were associated with negative behaviors, such as not coping well with losing, damaging equipment, and disrespectful behavior towards the judge and others. Bunketorp Käll et al. [67] found a positive effect on conduct problems among a subgroup of 4–6‐graders, while Mills et al. [78] reported increased conduct problems. One study found a positive effect on hyperactivity among a subgroup of 4–6‐grader girls [67], but two other studies found no effect on hyperactivity [69, 78]. Carter et al. [72] found no effect on externalizing symptoms, while Tennfjord et al. [73] reported no effect at three time points and a negative effect at one time point. Furthermore, Mills et al. [78] reported a positive effect on self‐injury/stereotypic behavior, and no effect on self‐isolated/ritualistic or irritable behavior.

#### 3.4.2 Mental Well‐Being

Mental well‐being was assessed in 23 studies (17 quantitative, four qualitative, and three mixed methods) using 18 different instruments and interviews. Various perspectives of mental well‐being were examined, including overall mental well‐being, psychosocial health‐related quality of life (HRQoL), psychological QoL, self‐esteem, self‐worth, self‐concept, self‐confidence, self‐efficacy, happiness, sense of coherence, resilience, and positive youth development. Ten studies (five quantitative, three qualitative, and two mixed methods) found a positive effect on at least one mental well‐being outcome, while 12 studies (11 quantitative and one mixed methods) reported no effect. One quantitative study reported mixed results. The numerical data from quantitative studies concerning mental well‐being is provided in Table S3 of Supporting Information S2. Two studies reported a positive effect on psychological/psychosocial QoL [56, 79], while three studies found no effect [80, 81, 82]. Four studies reported a positive effect on overall mental well‐being [83, 84, 85, 86], while four studies reported no effect [67, 68, 71, 87]. One study reported a negative effect on psychological well‐being among the subgroup of students who were most active at baseline [59]. However, in the same study, teachers reported that the intervention improved the well‐being among their students [60]. Two studies reported a positive effect on happiness [63, 64]. Three studies found no effect on self‐esteem [61, 62, 88, 89], while five studies found positive effects on self‐esteem [90], self‐worth [58], self‐confidence [64], self‐efficacy [84, 90], and self‐concept [91]. Sense of coherence, resilience, and positive youth development were each investigated in one study. No effect was found on any of these outcomes [68, 69, 92].

#### 3.4.3 Social Well‐Being

Social well‐being was assessed in 14 studies (seven quantitative, four qualitative, and three mixed methods) using nine different instruments and interviews. Social well‐being was examined from both positive and negative perspectives, including social behavior, social functioning, social competence, social interactions, social QoL, social support, relations to peers, and peer problems. Ten studies (three quantitative, four qualitative, and three mixed methods) found a positive effect on at least one social well‐being outcome, while four quantitative studies reported no effect. The numerical data from quantitative studies concerning social well‐being is provided in Table S4 of Supporting Information S2. Two studies found a positive effect on social behavior [78, 86], one found a positive effect on prosocial behavior among a subgroup of boys [69], and one study found a positive effect on prosocial peer affiliation, but no effect on prosocial behavior [93]. Two studies found no effect on prosocial behavior [67, 70]. Van Dijk‐Wesselius et al. [70] reported decreased peer problems, and Nathan et al. [69] reported decreased peer problems among a subgroup of boys. However, Bunketorp Käll et al. [67] found no effect on peer problems. Five studies reported improved social interactions [64, 65, 69, 77, 84], while one study found no effect [91]. Furthermore, Ryom et al. [91] reported an improved supportive social environment, and van Dijk‐Wesselius et al. [70] found improved social support. One study reported no effect on social QoL [79], and two studies found no effect on social functioning/competence [58, 66].

#### 3.4.4 Type of Physical Activity Promotion and Effects on Mental Health

PA promotion included permanent environmental modifications in five studies (three quantitative and two qualitative). Environmental modifications targeted to increase PA among students included new walking tracks, drop‐off zones around schools to encourage children to walk the remaining distance, playground constructions, greening the school yard, and dance mat systems. All five studies reported a positive effect on at least one mental health outcome. Allender et al. [56] found a positive effect on psychosocial HRQoL, and Azevedo et al. [83] reported improved psychological well‐being. Furthermore, Hamer et al. [84] found positive repercussions on well‐being, social interactions, and perceived self‐efficacy. Van Dijk‐Wesselius et al. [70] reported a positive effect on peer problems and social support, and Baker et al. [76] reported fewer behavioral problems.

Seven studies (four quantitative and three mixed methods) included PA promotion delivered by an external actor outside the school staff. PA promotion included additional PE lessons, a military ethos intervention, football training, Latino dance, nautical activities, and extracurricular sports. Six of the studies (three quantitative and three mixed methods) found a positive effect on at least one mental health outcome, while one quantitative study reported mixed results. Bunketorp Käll et al. [67] found positive effects on conduct problems among a subgroup of 4–6 graders and hyperactivity among a subgroup of 4–6 grade girls, while Mills et al. [78] reported a negative effect on conduct problems. However, Mills et al. [78] found positive effects on positive social behavior and self‐injury/stereotypic behavior. Nathan et al. [69] reported fewer peer problems and improved prosocial behavior, and Villarreal & Gonzalez [93] found a positive effect on prosocial peer affiliation. Furthermore, Rocher et al. [86] reported benefits to mental health and well‐being, social behavior, and sense of community, and Ryom et al. [91] reported improved self‐concept and a more supportive social environment. Zhou et al. [90] found a positive effect on self‐efficacy and self‐esteem.

Ten studies (nine quantitative and one qualitative) included PA promotion delivered by school staff following a specific, predefined protocol. PA promotion included soccer, additional PE classes, in‐class activities, active outdoor lessons, recess/lunchtime activities, active breaks, school sports teams, physically active homework, and education. Four quantitative studies found a positive effect on at least one mental health outcome, while four quantitative studies found no effect. Two studies (one quantitative and one qualitative) reported mixed results. Positive effects were found on internalizing symptoms [72], anxiety [74], empathy, and social connections [77]. Christiansen et al. [58] found improved self‐worth, while Lubans et al. [89] reported no effect on self‐esteem. Kvalø & Natlandsmyr [85] reported improved psychological well‐being, while Christiansen et al. [59] found a negative effect on psychological well‐being among the subgroup of those who were most active at baseline. One study reported a positive effect on psychosocial QoL [79], while two studies found no effect [81, 82]. Furthermore, Woodgate & Sigurdson [92] found no effect on positive youth development, and Holt et al. [77] reported increased negative behavior during the lunchtime sports that were part of the PA program.

Nine quantitative studies included PA promotion delivered by school staff without a specific protocol. Interventions included training, materials, and equipment to support school staff in developing and implementing PA promotion at school. Seven studies reported no effect on mental health [61, 62, 66, 68, 71, 80, 87, 88]. One study [75] found a positive effect on depressive symptoms favoring one intervention school with the objective of increasing mental well‐being through the promotion of healthy eating and PA, compared to the other two intervention schools with the objectives of increasing PA at school and increasing active transport to and from school. However, depressive symptoms were not analyzed comparing the intervention and control groups. One study reported no effect on internalizing symptoms at three time points, but a negative effect at one time point, as well as no effect on externalizing symptoms at three time points, but a negative effect at one time point [73].

## 4 Discussion

To our knowledge, this is the first systematic review investigating the effects of long‐term school‐based PA promotion on mental health among children and adolescents. In addition, this is the first review considering externalizing symptoms alongside internalizing symptoms, mental well‐being, and social well‐being. In total, 38 articles describing 34 studies were included. The findings showed benefits for social well‐being, while mixed results on internalizing symptoms, externalizing symptoms, and mental well‐being were found. Regarding the type of PA promotion, environmental modifications and PA promotion delivered by an actor external to the school staff were found to be effective. PA promotion delivered by school staff without any specific protocol had no effect on mental health, while PA promotion delivered by school staff following a specific and predefined protocol showed mixed results. The results are based on both quantitative and qualitative findings, which were consistent with each other. The results did not differ based on whether the aim of the PA promotion was to improve mental health or whether mental health was measured as a secondary outcome.

### 4.1 Internalizing and Externalizing Symptoms

Findings regarding internalizing symptoms were consistent with the results of the previous systematic review and meta‐analysis investigating the effect of school‐related PA interventions on mental health. Andermo et al. [15] found a positive effect on anxiety, but no effect on internalizing symptoms in general. Similarly, in this review, two studies on anxiety showed positive findings, but the results regarding internalizing symptoms in general were mixed. Furthermore, in the recent systematic review, the effect of PA interventions in school and community settings on psychological ill‐being remained unclear [16].

Results concerning externalizing symptoms in this review were mixed. Previous reviews of the topic have not elucidated the effect of school‐based PA on externalizing symptoms. In a meta‐analytic review, not limited to the school setting, a positive effect of PA interventions on externalizing problems was found [94]. However, the association between PA and externalizing symptoms is complex, as Asfour et al. [95] found that a low level of PA as well as a greater level of unorganized PA may increase symptoms. Furthermore, a recent study among young adolescents with difficult temperaments and exposed to family adversity reported the complexity of the association between PA and internalizing and externalizing problems [96].

Mixed results regarding internalizing and externalizing symptoms may be due to the heterogeneity of study designs, the instruments used for measuring symptoms, and PA promotion actions. Although all studies included long‐term PA promotion, there was still variation in the duration, content, and implementation of PA promotion, as well as in the amount and intensity of PA. Further research with more consistent methods and instruments is needed on the effects of school‐based PA on internalizing and externalizing symptoms.

### 4.2 Mental Well‐Being

Findings regarding mental well‐being were mixed, which is consistent with the results of the previous review [17]. However, two other reviews reported the positive effect of PA interventions in school and community settings on mental well‐being [15, 16]. The contrary results may be due to the shorter duration of the intervention. In this review, only PA promotion actions having a duration of at least 9 months were included. In previous review articles, longer interventions were relatively rare. In a review by Andermo et al. [15], only seven of 31 interventions had a duration of at least 9 months, and only two of them found an effect on mental well‐being. Similarly, only three of 27 interventions included in the review by Hale et al. [16] had a duration of at least 9 months, and none of them found an effect on mental well‐being. It has been shown that duration biases are common in intervention studies, with shorter durations more likely to yield effective results [97].

In addition to the diversity in study designs and PA promotion actions, mixed results concerning mental well‐being may be particularly attributed to the wide array of measurement instruments. Some instruments measured mental well‐being broadly, while others focused narrowly on specific aspects of mental well‐being. However, further research is essential to determine how to effectively promote mental well‐being in children and adolescents in the long run.

### 4.3 Social Well‐Being

A previous review investigating the effectiveness of the interventions using PA for the promotion of social competence at school reported a positive effect on social competence. However, only four of 29 studies included a long‐term intervention with a duration of at least 9 months [18]. The findings of this review complemented previous results showing the effectiveness of long‐term PA promotion on social well‐being. The positive effect of school‐based PA promotion on social well‐being may be mediated through improved social skills, as in a previous systematic review, physical education and sports were found to enhance the social skills of children and adolescents [98]. Furthermore, PA promotion may strengthen community spirit, foster a sense of belonging, and create shared experiences of achievement, which can influence the social well‐being of the students.

The impact of school‐based PA promotion on social well‐being is a significant finding, as social well‐being is associated with both physical and mental health and well‐being, and impairments in social well‐being can have severe consequences. School‐based social inclusion and exclusion have been found to predict loneliness, mental health problems, and subjective well‐being [99]. Furthermore, Bania et al. [100] reported that peer problems among adolescents were associated with later not engaged in education, employment, or training (NEET) status. A review of reviews investigated the public health implications of social isolation, reporting poorer mental health, increased all‐cause mortality, and cardiovascular disease [101]. Conversely, positive relations with peers in adolescence are associated with mental well‐being in early adulthood [102].

### 4.4 Type of Physical Activity Promotion and Effects on Mental Health

In this review, environmental modifications at school were found to be effective on students' mental health. The finding is consistent with the previous review reporting that schoolyard greening had a positive effect on students' socioemotional health [103]. Environmental modifications at school are a promising approach to promote the health and well‐being of children and adolescents, as a previous review has shown that such modifications are effective in increasing PA [104], complementing the findings of this review on mental health.

PA promotion delivered by an external actor outside the school staff was found to be effective, while PA promotion delivered by school staff following a specific program showed mixed results, and PA promotion delivered by school staff without any specific program showed no effect on mental health. These results may be explained by the curricular demands and lack of knowledge, self‐efficacy, time, and space to promote PA among teachers. In a systematic review investigating the barriers and facilitators to the implementation of PA policies in schools, the most frequent barriers included curriculum demands, as PA is considered a lower priority compared to other subjects, a lack of time in the curriculum, and a lack of teacher expertise delivering PA [41]. Consistently, Dyrstad et al. [105] found that the main barriers to planning physically active lessons were a lack of knowledge and time. Furthermore, in the study by Martyn et al. [106], the barriers to implementing 20 min of daily PA were space and time constraints, inadequate training, student behavioral issues, and the teacher's low self‐efficacy. Based on the findings of this review, it can be recommended that schools should have a dedicated individual (e.g., sports instructor, health and wellness coach, school personal trainer) with sufficient expertise and relatively few or no curricular demands to promote PA among students. This is further supported by the results of a previous meta‐analysis that found significant improvements in educational outcomes when physically active lessons were delivered by recruited personnel, while physically active lessons delivered by a classroom teacher had no effect [107]. Furthermore, in a previous review, the most frequent facilitators to the implementation of PA policies in schools included having a PA specialist in the school [41], and wider stakeholders are mentioned as one of the essential groups for facilitating whole‐school PA implementation in the Creating Active Schools Framework [108].

### 4.5 Quality of Evidence

Regarding mental health outcomes, the main factor compromising the quality of evidence is that only about half of the studies were of good quality (≤ 1 quality concern according to the MMAT assessment). In the other half, multiple (≥ 2) quality concerns were identified, which may reduce the reliability of the findings. However, across all mental health outcomes, the evidence is strengthened by the fact that it is based on both quantitative and qualitative data, reported by children themselves as well as parents and teachers. However, in relation to externalizing symptoms and social well‐being, it is important to note that the lack of randomized study designs may affect the overall quality of the evidence. Furthermore, as the findings concerning these two outcomes primarily focus on children and adolescents over the age of 10, they should be interpreted with caution when considering younger age groups. Detailed assessments for each mental health outcome are provided in Table S5 of Supporting Information S2.

There were differences in the quality of evidence across different types of PA promotion. Regarding permanent environmental modifications, the evidence is mainly based on good‐quality studies employing various study designs, with both quantitative and qualitative data supporting the findings. However, all participants in the included studies were under the age of 14, and therefore the results cannot be generalized to older age groups. The evidence concerning PA promotion delivered by an external actor outside the school staff should be interpreted with particular caution, as limitations in study quality weaken the strength of the evidence. Only one of the seven studies was of good quality, while the remaining six exhibited multiple quality concerns. Furthermore, the evidence in this category is based solely on non‐randomized designs. For PA promotion delivered by school staff following a specific, predefined protocol, no major weaknesses in the evidence were identified. However, only half of the studies were of good quality. The evidence regarding PA promotion delivered by school staff without a specific protocol is based exclusively on quantitative data and relies almost entirely on self‐reported data, which should be taken into account when interpreting the findings. Furthermore, the results mainly concern children over the age of 10 and should therefore be interpreted with caution when considering younger age groups. Detailed assessments for each type of PA promotion are provided in Table S6 of Supporting Information S2.

### 4.6 Possible Adverse Effects

When designing school‐based PA interventions, it is important to acknowledge that individuals experience PA differently. Some children may feel uncomfortable in physically active situations, and, for example, children with overweight may be at risk of stigmatization [109]. However, such themes did not emerge in the studies included in this review, including qualitative interviews. This may be due to the nature of the interventions, which were generally easy to approach and rarely competitive, allowing for low‐threshold participation and potentially increasing the likelihood of positive experiences. In addition, unlike in PE, students' performance in PA was not evaluated, which may have enabled carefree participation according to one's own abilities and motivation.

Some negative effects were also reported in the studies included in this review. Mills et al. [78] reported increased conduct problems in the intervention group that participated in a military ethos PA intervention. Holt et al. [77] described negative behaviors related to competitiveness during lunchtime intramural sports, including indoor soccer, floor hockey, and basketball. Tennfjord et al. [73] reported increased psychosocial problems following an intervention that added 45 min of PA each day, replacing traditional desk‐based learning with physical tasks.

School‐based PA promotion should be carefully designed, taking into account potential negative effects and special needs. Previous research has shown that adapted pedagogical strategies in PE are necessary when working with, for example, physically or intellectually disabled students [110]. Therefore, when planning school‐based PA promotion, it is essential to ensure that the activities are inclusive and do not stigmatize any participants. Further research is needed to explore this perspective in more depth.

### 4.7 Strengths and Limitations

This review has several strengths. The review is reported using the PRISMA guidelines, and the protocol for this review was pre‐registered in the PROSPERO database. Considering the nature of the school as a non‐clinical research setting, comprehensive inclusion criteria, including various study designs and intervention formats, were utilized. Furthermore, a comprehensive definition of mental health, including mental health problems, mental well‐being, and social well‐being, enabled a novel review of the topic. An extensive search strategy, including various positive and negative search terms of mental health, was tested and developed, and the comprehensive literature search was conducted across six databases, supplemented by backward and forward snowballing. The study selection process was performed by two independent researchers.

The review has several limitations as well. First, by restricting the language of the included publications to English, relevant studies published in other languages may have been missed. Second, due to funding and time constraints, quality assessment and data extraction were performed by one independent researcher only. However, the accuracy of the results has been verified by the other authors. Third, due to the considerable heterogeneity in study designs, the sample sizes, PA promotion actions, control and comparison groups, and instruments used across the studies, the synthesis of the results was limited to a narrative approach. Fourth, as some included studies were based on a multi‐component program including PA promotion along with other components, it is challenging to isolate the specific contribution of PA promotion to the results in these cases. However, the results remained robust in the sensitivity analyses including only studies with single‐component programs focused solely on PA promotion. Finally, due to the mixed methodological quality of the included studies, the findings should be interpreted with caution.

### 4.8 Future Research Directions

Research evidence regarding the association between school‐based PA promotion and mental health among children and adolescents is inadequate and partly inconsistent. The results obtained from this and previous reviews on internalizing symptoms, externalizing symptoms, and mental well‐being are mixed; thus, the evidence remains unclear. Future research is needed to determine whether these aspects of mental health can be promoted through school‐based PA promotion. Although the results regarding social well‐being are consistent, it should be noted that they are based on a relatively small number of studies. To strengthen the evidence, further research is needed on the associations between school‐based PA promotion and social well‐being as well. In this review, it was not possible to investigate potential differences in associations between school‐based PA promotion and mental health among different subgroups, as only a few studies reported results related to various subgroups. Further research is needed to investigate whether there are differences in these associations among various subgroups, such as different sexes, age groups, or socioeconomic backgrounds, as well as whether there are differences in the associations among individuals who engage in PA during their leisure time and those who do not. Furthermore, in the future, it would be important to elucidate the mechanisms through which various interventions affect mental health and why interventions led by teachers are less effective than those led by external actors.

## 5 Conclusions

The findings indicate that school‐based PA promotion is linked to enhanced social well‐being among children and adolescents and may thus contribute to the promotion of social skills and positive social relations. Regarding the type of PA promotion, environmental modifications and PA promotion delivered by an external actor outside the school staff were found to be beneficial for mental health. Considering the promising results of this review, and the well‐established benefits of PA on the overall health of children and adolescents, it can be recommended that schools invest in promoting PA. However, when designing PA promotion initiatives, it is important to ensure that the implementation is approachable and inclusive in order to prevent potential adverse effects, such as the marginalization or stigmatization of certain student groups. Furthermore, since teachers and school staff play a central role in shaping school culture and implementing PA promotion, more research is needed to understand why teacher‐delivered interventions may not appear to be effective.

## 6 Perspective

The physical inactivity of children and adolescents has already been identified as a serious public health concern [34]. Over the past decades, PA among children and adolescents has decreased, while mental health problems have concurrently increased [1, 2, 3, 4, 5, 6, 7]. The benefits of adequate PA on mental health among children and adolescents are well‐known [8, 9, 10]. School is an ideal setting for health promotion [13, 14], and the need for long‐term interventions has been recognized [40, 44, 45, 46, 47]. Consequently, there is growing attention on developing school‐based PA promotion to support mental health. To our knowledge, this is the first systematic review investigating the effects of long‐term school‐based PA promotion on mental health among children and adolescents. This review also provides new insights into the types of PA promotion in schools that contribute to mental health benefits. As a result, we found a positive effect on social well‐being. The beneficial types of PA promotion on mental health were environmental modifications and PA promotion delivered by an external actor outside the school staff. Based on the results, it is recommended that schools invest in promoting PA to enhance the social well‐being of children and adolescents and prevent social exclusion in the future.

## Conflicts of Interest

The authors declare no conflicts of interest.

## Supporting information


**Appendix S1:** sms70150‐sup‐0001‐AppendixS1.docx.

**Appendix S2:** sms70150‐sup‐0002‐AppendixS2.docx.

## Data Availability

Data sharing not applicable to this article as no datasets were generated or analysed during the current study.

## References

[1] S. Bremberg , “Mental Health Problems Are Rising More in Swedish Adolescents Than in Other Nordic Countries and the Netherlands,” Acta Paediatrica 104, no. 10 (2015): 997–1004, 10.1111/apa.13075. 26096198

[2] Centers for Disease Control and Prevention , “Youth Risk Behavior Survey Data Summary & Trends Report: 2011–2021,” (U.S. Department of Health and Human Services, 2023).

[3] K. M. Keyes , D. Gary , P. M. O'Malley , A. Hamilton , and J. Schulenberg , “Recent Increases in Depressive Symptoms Among US Adolescents: Trends From 1991 to 2018,” Social Psychiatry and Psychiatric Epidemiology 54, no. 8 (2019): 987–996, 10.1007/s00127-019-01697-8. 30929042 PMC7015269

[4] J. Pitchforth , K. Fahy , T. Ford , M. Wolpert , R. M. Viner , and D. S. Hargreaves , “Mental Health and Well‐Being Trends Among Children and Young People in the UK, 1995–2014: Analysis of Repeated Cross‐Sectional National Health Surveys,” Psychological Medicine 49, no. 8 (2019): 1275–1285, 10.1017/S0033291718001757. 30201061 PMC6518382

[5] I. E. Thorisdottir , B. B. Asgeirsdottir , R. Sigurvinsdottir , J. P. Allegrante , and I. D. Sigfusdottir , “The Increase in Symptoms of Anxiety and Depressed Mood Among Icelandic Adolescents: Time Trend Between 2006 and 2016,” European Journal of Public Health 27, no. 5 (2017): 856–861, 10.1093/eurpub/ckx111. 28957485

[6] K. Wiens , A. Bhattarai , P. Pedram , et al., “A Growing Need for Youth Mental Health Services in Canada: Examining Trends in Youth Mental Health From 2011 to 2018,” Epidemiology and Psychiatric Sciences 29 (2020): e115, 10.1017/S2045796020000281. 32299531 PMC7214527

[7] S. A. Conger , L. P. Toth , C. Cretsinger , et al., “Time Trends in Physical Activity Using Wearable Devices: A Systematic Review and Meta‐Analysis of Studies From 1995 to 2017,” Medicine and Science in Sports and Exercise 54, no. 2 (2022): 288–298, 10.1249/MSS.0000000000002794. 34559725

[8] S. J. H. Biddle , S. Ciaccioni , G. Thomas , and I. Vergeer , “Physical Activity and Mental Health in Children and Adolescents: An Updated Review of Reviews and an Analysis of Causality,” Psychology of Sport and Exercise 42 (2019): 146–155, 10.1016/j.psychsport.2018.08.011.

[9] F. Recchia , J. D. K. Bernal , D. Y. Fong , et al., “Physical Activity Interventions to Alleviate Depressive Symptoms in Children and Adolescents: A Systematic Review and Meta‐Analysis,” JAMA Pediatrics 177, no. 2 (2023): 132–140, 10.1001/jamapediatrics.2022.5090. 36595284 PMC9857695

[10] M. Rodriguez‐Ayllon , C. Cadenas‐Sánchez , F. Estévez‐López , et al., “Role of Physical Activity and Sedentary Behavior in the Mental Health of Preschoolers, Children and Adolescents: A Systematic Review and Meta‐Analysis,” Sports Medicine 49, no. 9 (2019): 1383–1410, 10.1007/s40279-019-01099-5. 30993594

[11] H. Zhao , N. Wu , E. A. Haapala , and Y. Gao , “Association Between Meeting 24‐h Movement Guidelines and Health in Children and Adolescents Aged 5–17 Years: A Systematic Review and Meta‐Analysis,” Frontiers in Public Health 12 (2024): 1351972, 10.3389/fpubh.2024.1351972. 38774055 PMC11106490

[12] B. Singh , H. Bennett , A. Miatke , et al., “Systematic Umbrella Review and Meta‐Meta‐Analysis: Effectiveness of Physical Activity in Improving Depression and Anxiety in Children and Adolescents,” Journal of the American Academy of Child and Adolescent Psychiatry S0890‐8567, no. 25 (2025): 204–208, 10.1016/j.jaac.2025.04.007. 40239946

[13] M. Pulimeno , P. Piscitelli , S. Colazzo , A. Colao , and A. Miani , “School as Ideal Setting to Promote Health and Wellbeing Among Young People,” Health Promotion Perspective 10, no. 4 (2020): 316–324, 10.34172/hpp.2020.50. PMC7723000 33312927

[14] E. M. F. , van van Sluijs , U. Ekelund , I. Crochemore‐Silva , et al., “Physical Activity Behaviours in Adolescence: Current Evidence and Opportunities for Intervention,” Lancet 398, no. 10298 (2021): 429–442, 10.1016/S0140-6736(21)01259-9. 34302767 PMC7612669

[15] S. Andermo , M. Hallgren , T. T. D. Nguyen , et al., “School‐Related Physical Activity Interventions and Mental Health Among Children: A Systematic Review and Meta‐Analysis,” Sports Medicine ‐ Open 6, no. 1 (2020): 25, 10.1186/s40798-020-00254-x. 32548792 PMC7297899

[16] G. E. Hale , L. Colquhoun , D. Lancastle , N. Lewis , and P. J. Tyson , “Physical Activity Interventions for the Mental Health of Children: A Systematic Review,” Child: Care, Health and Development 49, no. 2 (2023): 211–229, 10.1111/cch.13048. 35995884

[17] R. Rafferty , G. Breslin , D. Brennan , and D. Hassan , “A Systematic Review of School‐Based Physical Activity Interventions on Children's Wellbeing,” International Review of Sport and Exercise Psychology 9, no. 1 (2016): 215–230, 10.1080/1750984X.2016.1164228.

[18] I. Schüller and Y. Demetriou , “Physical Activity Interventions Promoting Social Competence at School: A Systematic Review,” Educational Research Review 25 (2018): 39–55, 10.1016/j.edurev.2018.09.001.

[19] World Health Organization , World Mental Health Report: Transforming Mental Health for All (World Health Organization, 2022).

[20] R. Tennant , L. Hiller , R. Fishwick , et al., “The Warwick‐Edinburgh Mental Well‐Being Scale (WEMWBS): Development and UK Validation,” Health and Quality of Life Outcomes 5, no. 1 (2007): 63, 10.1186/1477-7525-5-63. 18042300 PMC2222612

[21] T. J. Huberty , “Emotional and Behavioral Problems, Students With,” in Encyclopedia of Applied Psychology, ed. C. D. Spielberger (Elsevier, 2004), 723–730, 10.1016/B0-12-657410-3/00791-1.

[22] M. W. Gallagher , S. J. Lopez , and K. J. Preacher , “The Hierarchical Structure of Well‐Being,” Journal of Personality 77, no. 4 (2009): 1025–1050, 10.1111/j.1467-6494.2009.00573.x. 19558444 PMC3865980

[23] C. L. M. Keyes , “Mental Illness and/or Mental Health? Investigating Axioms of the Complete State Model of Health,” Journal of Consulting and Clinical Psychology 73, no. 3 (2005): 539–548, 10.1037/0022-006X.73.3.539. 15982151

[24] K. K. Sarasjärvi , M. Elovainio , K. Appelqvist‐Schmidlechner , P. Solin , N. Tamminen , and S. Therman , “Exploring the Structure and Psychometric Properties of the Warwick‐Edinburgh Mental Well‐Being Scale (WEMWBS) in a Representative Adult Population Sample,” Psychiatry Research 328 (2023): 115465, 10.1016/j.psychres.2023.115465. 37708805

[25] C. L. M. Keyes and J. Annas , “Feeling Good and Functioning Well: Distinctive Concepts in Ancient Philosophy and Contemporary Science,” Journal of Positive Psychology 4, no. 3 (2009): 197–201, 10.1080/17439760902844228.

[26] R. M. Ryan and E. L. Deci , “On Happiness and Human Potentials: A Review of Research on Hedonic and Eudaimonic Well‐Being,” Annual Review of Psychology 52 (2001): 141–166, 10.1146/annurev.psych.52.1.141. 11148302

[27] M. Solmi , J. Radua , M. Olivola , et al., “Age at Onset of Mental Disorders Worldwide: Large‐Scale Meta‐Analysis of 192 Epidemiological Studies,” Molecular Psychiatry 27, no. 1 (2022): 281–295, 10.1038/s41380-021-01161-7. 34079068 PMC8960395

[28] C. L. M. Keyes , S. S. Dhingra , and E. J. Simoes , “Change in Level of Positive Mental Health as a Predictor of Future Risk of Mental Illness,” American Journal of Public Health 100, no. 12 (2010): 2366–2371, 10.2105/AJPH.2010.192245. 20966364 PMC2978199

[29] World Health Organization , “Promoting Mental Health: Concepts, Emerging Evidence, Practice: Report of the World Health Organization,” (2005), https://www.who.int/publications‐detail‐redirect/9241562943.

[30] Regional Health–Europe TL , “Protecting the Mental Health of Youth,” Lancet Regional Health ‐ Europe 12 (2022): 100306, 10.1016/j.lanepe.2021.100306. 35059684 PMC8758917

[31] World Health Organization , “Guidelines on Mental Health Promotive and Preventive Interventions for Adolescents,” (2020), https://www.who.int/publications‐detail‐redirect/9789240011854. 33301276

[32] C. J. Caspersen , K. E. Powell , and G. M. Christenson , “Physical Activity, Exercise, and Physical Fitness: Definitions and Distinctions for Health‐Related Research,” Public Health Reports 100, no. 2 (1985): 126–131. 3920711 PMC1424733

[33] F. C. Bull , S. S. Al‐Ansari , S. Biddle , et al., “World Health Organization 2020 Guidelines on Physical Activity and Sedentary Behaviour,” British Journal of Sports Medicine 54, no. 24 (2020): 1451–1462, 10.1136/bjsports-2020-102955. 33239350 PMC7719906

[34] S. Aubert , J. D. Barnes , I. Demchenko , et al., “Global Matrix 4.0 Physical Activity Report Card Grades for Children and Adolescents: Results and Analyses From 57 Countries,” Journal of Physical Activity & Health 19, no. 11 (2022): 700–728, 10.1123/jpah.2022-0456. 36280233

[35] M. A. Farooq , K. N. Parkinson , A. J. Adamson , et al., “Timing of the Decline in Physical Activity in Childhood and Adolescence: Gateshead Millennium Cohort Study,” British Journal of Sports Medicine 52, no. 15 (2018): 1002–1006, 10.1136/bjsports-2016-096933. 28288966 PMC6204977

[36] R. Guthold , G. A. Stevens , L. M. Riley , and F. C. Bull , “Global Trends in Insufficient Physical Activity Among Adolescents: A Pooled Analysis of 298 Population‐Based Surveys With 1·6 Million Participants,” Lancet Child Adolesc Health 4, no. 1 (2020): 23–35, 10.1016/S2352-4642(19)30323-2. 31761562 PMC6919336

[37] United Nations Educational, Scientific and Cultural Organization , Global Education Monitoring Report 2023: Technology in Education—A Tool on Whose Terms? (United Nations Educational, Scientific and Cultural Organization, 2023), 10.18356/9789210028660.

[38] D. A. Farbman , The Case for Improving and Expanding Time in School: A Review of Key Research and Practice (National Center on Time & Learning, 2012).

[39] K. Milton , N. Cavill , A. Chalkley , et al., “Eight Investments That Work for Physical Activity,” Journal of Physical Activity and Health 18 (2021): 625–630, 10.1123/jpah.2021-0112. 33984836

[40] C. L. M. Keyes , “Promoting and Protecting Positive Mental Health: Early and Often Throughout the Lifespan,” in Mental Well‐Being: International Contributions to the Study of Positive Mental Health, ed. C. L. M. Keyes (Springer Netherlands, 2013), 3–28, 10.1007/978-94-007-5195-8_1.

[41] N. Nathan , B. Elton , M. Babic , et al., “Barriers and Facilitators to the Implementation of Physical Activity Policies in Schools: A Systematic Review,” Preventive Medicine 107 (2018): 45–53, 10.1016/j.ypmed.2017.11.012. 29155228

[42] S. Peters , “Qualitative Research Methods in Mental Health,” Evidence‐Based Mental Health 13, no. 2 (2010): 35–40, 10.1136/ebmh.13.2.35. 21856603

[43] M. L. Nelson and S. M. Quintana , “Qualitative Clinical Research With Children and Adolescents,” Journal of Clinical Child and Adolescent Psychology 34, no. 2 (2005): 344–356, 10.1207/s15374424jccp3402_14. 15901235

[44] M. M. Barry , A. M. Clarke , R. Jenkins , and V. Patel , “A Systematic Review of the Effectiveness of Mental Health Promotion Interventions for Young People in Low and Middle Income Countries,” BMC Public Health 13, no. 1 (2013): 835, 10.1186/1471-2458-13-835. 24025155 PMC3848687

[45] R. F. Catalano , M. L. Berglund , J. A. M. Ryan , H. S. Lonczak , and J. D. Hawkins , “Positive Youth Development in the United States: Research Findings on Evaluations of Positive Youth Development Programs,” Annals of the American Academy of Political and Social Science 591 (2004): 98–124.

[46] J. Wells , J. Barlow , and S. Stewart‐Brown , “A Systematic Review of Universal Approaches to Mental Health Promotion in Schools,” Health Education 103, no. 4 (2003): 197–220, 10.1108/09654280310485546.

[47] A. Drouka , D. Brikou , C. Causeret , et al., “Effectiveness of School‐Based Interventions in Europe for Promoting Healthy Lifestyle Behaviors in Children,” Children 10, no. 10 (2023): 1676, 10.3390/children10101676. 37892339 PMC10605522

[48] M. J. Page , J. E. McKenzie , P. M. Bossuyt , et al., “The PRISMA 2020 Statement: An Updated Guideline for Reporting Systematic Reviews,” British Medical Journal 372 (2021): n71, 10.1136/bmj.n71. 33782057 PMC8005924

[49] Q. N. Hong , A. Gonzalez‐Reyes , and P. Pluye , “Improving the Usefulness of a Tool for Appraising the Quality of Qualitative, Quantitative and Mixed Methods Studies, the mixed methods appraisal tool (mmat),” Evaluation Clinical Practice 24, no. 3 (2018): 459–467, 10.1111/jep.12884. 29464873

[50] Q. N. Hong , P. Pluye , S. Fàbregues , et al., “Improving the Content Validity of the Mixed Methods Appraisal Tool: A Modified e‐Delphi Study,” Journal of Clinical Epidemiology 111 (2019): 49–59.e1, 10.1016/j.jclinepi.2019.03.008. 30905698

[51] R. Q. Souto , V. Khanassov , Q. N. Hong , P. L. Bush , I. Vedel , and P. Pluye , “Systematic Mixed Studies Reviews: Updating Results on the Reliability and Efficiency of the Mixed Methods Appraisal Tool,” International Journal of Nursing Studies 52, no. 1 (2015): 500–501, 10.1016/j.ijnurstu.2014.08.010. 25241931

[52] R. Ryan , “Cochrane Consumers and Communication Review Group. ‘Cochrane Consumers and Communication Review Group: Data Synthesis and Analysis’” (2019), http://cccrg.cochrane.org.

[53] L. Lizarondo , H. Loveday , S. Salmond , et al., “Systems for Assessing the Certainty or Confidence of Evidence in Healthcare: A Scoping Review Protocol,” JBI Evidence Synthesis 23, no. 9 (2025): 1817–1823, 10.11124/JBIES-24-00556. 40269553

[54] G. I. Niño Cruz , J. J. Yepes‐Nuñez , C. Valli , A. Garcia Sierra , and E. Besnier , GRADE Mixed Methods Approach in Systematic Reviews and Guideline Development: An Exploratory Survey (OSF, 2024), 10.17605/OSF.IO/9BGEH.

[55] N. Bray , N. Kolehmainen , J. McAnuff , et al., “Rationale for the Chosen Approach to Assessing the Certainty in the Body of Evidence in the Integrated Mixed‐Methods Evidence Synthesis,” in Powered Mobility Interventions for Very Young Children With Mobility Limitations to Aid Participation and Positive Development: The EMPoWER Evidence Synthesis (NIHR Journals Library, 2020), https://www.ncbi.nlm.nih.gov/books/NBK563098/. 10.3310/hta24500 PMC7681349 33078704

[56] S. Allender , L. Orellana , N. Crooks , et al., “Four‐Year Behavioral, Health‐Related Quality of Life, and BMI Outcomes From a Cluster Randomized Whole of Systems Trial of Prevention Strategies for Childhood Obesity,” Obesity 29, no. 6 (2021): 1022–1035, 10.1002/oby.23130. 33950583 PMC8251751

[57] J. Jacobs , C. Strugnell , S. Allender , et al., “The Impact of a Community‐Based Intervention on Weight, Weight‐Related Behaviours and Health‐Related Quality of Life in Primary School Children in Victoria, Australia, According to Socio‐Economic Position,” BMC Public Health 21, no. 1 (2021): 2179, 10.1186/s12889-021-12150-4. 34837974 PMC8627608

[58] L. B. Christiansen , P. Lund‐Cramer , R. Brondeel , S. Smedegaard , A. D. Holt , and T. Skovgaard , “Improving Children's Physical Self‐Perception Through a School‐Based Physical Activity Intervention: The Move for Well‐Being in School Study,” Mental Health and Physical Activity 14 (2018): 31–38, 10.1016/j.mhpa.2017.12.005.

[59] L. B. Christiansen , R. Brondeel , P. Lund‐Cramer , S. Smedegaard , and T. Skovgaard , “Different Effects of a School‐Based Physical Activity Intervention on Health‐Related Quality of Life,” Applied Research in Quality of Life 17, no. 3 (2022): 1767–1785, 10.1007/s11482-021-10002-2.

[60] S. Smedegaard , R. Brondeel , L. B. Christiansen , and T. Skovgaard , “What Happened in the ‘Move for Well‐Being in School’: A Process Evaluation of a Cluster Randomized Physical Activity Intervention Using the RE‐AIM Framework,” International Journal of Behavioral Nutrition and Physical Activity 14, no. 1 (2017): 159, 10.1186/s12966-017-0614-8. 29145868 PMC5689205

[61] D. M. Harrington , M. J. Davies , D. H. Bodicoat , et al., “Effectiveness of the ‘Girls Active’ School‐Based Physical Activity Programme: A Cluster Randomised Controlled Trial,” International Journal of Behavioral Nutrition and Physical Activity 15, no. 1 (2018): 40, 10.1186/s12966-018-0664-6. 29695250 PMC5918764

[62] D. M. Harrington , M. J. Davies , D. Bodicoat , et al., “A School‐Based Intervention (‘Girls Active’) to Increase Physical Activity Levels Among 11‐ to 14‐Year‐Old Girls: Cluster RCT,” Public Health Research 7, no. 5 (2019): 1–162, 10.3310/phr07050. 30779533

[63] V. Jägerbrink , J. Glaser , and A. H. Östenberg , “Extracurricular Pulse Activities in School: Students' Attitudes and Experiences,” International Journal of Environmental Research and Public Health 19, no. 22 (2022): 15051, 10.3390/ijerph192215051. 36429770 PMC9691175

[64] A. Lazaridis , I. Syrmpas , C. Krommidas , and N. Digelidis , “Perceptions and Experiences After Participating in a Two‐Year Outdoor Adventure Programme,” Physical Culture and Sport Studies and Research 100, no. 1 (2023): 35–46, 10.2478/pcssr-2023-0017.

[65] T. Schirmer , A. Bailey , N. Kerr , A. Walton , L. Ferrington , and M. E. Cecilio , “Start Small and Let It Build; a Mixed‐Method Evaluation of a School‐Based Physical Activity Program, Kilometre Club,” BMC Public Health 23, no. 1 (2023): 137, 10.1186/s12889-022-14927-7. 36658556 PMC9850327

[66] C. Barnes , A. Hall , N. Nathan , et al., “Efficacy of a School‐Based Physical Activity and Nutrition Intervention on Child Weight Status: Findings From a Cluster Randomized Controlled Trial,” Preventive Medicine 153 (2021): 106822, 10.1016/j.ypmed.2021.106822. 34599925

[67] L. Bunketorp Käll , H. Malmgren , E. Olsson , T. Lindén , and M. Nilsson , “Effects of a Curricular Physical Activity Intervention on Children's School Performance, Wellness, and Brain Development,” Journal of School Health 85, no. 10 (2015): 704–713, 10.1111/josh.12303. 26331753

[68] L. Grillich , C. Kien , Y. Takuya , M. Weber , and G. Gartlehner , “Effectiveness Evaluation of a Health Promotion Programme in Primary Schools: A Cluster Randomised Controlled Trial,” BMC Public Health 16, no. 1 (2016): 679, 10.1186/s12889-016-3330-4. 27475339 PMC4967511

[69] S. Nathan , L. Kemp , A. Bunde‐Birouste , J. MacKenzie , C. Evers , and T. A. Shwe , ““We Wouldn't of Made Friends if We didn't Come to Football United”: The Impacts of a Football Program On Young People's Peer, Prosocial and Cross‐Cultural Relationships,” BMC Public Health 13, no. 1 (2013): 399, 10.1186/1471-2458-13-399. 23621898 PMC3649946

[70] J. E. van Dijk‐Wesselius , J. Maas , D. Hovinga , M. van Vugt , and A. E. van den Berg , “The Impact of Greening Schoolyards on the Appreciation, and Physical, Cognitive and Social–Emotional Well‐Being of Schoolchildren: A Prospective Intervention Study,” Landscape and Urban Planning 180 (2018): 15–26, 10.1016/j.landurbplan.2018.08.003.

[71] D. R. Lubans , J. J. Smith , N. Eather , et al., “Time‐Efficient Intervention to Improve Older Adolescents' Cardiorespiratory Fitness: Findings From the ‘Burn 2 Learn’ Cluster Randomised Controlled Trial,” British Journal of Sports Medicine 55, no. 13 (2021): 751–758, 10.1136/bjsports-2020-103277. PMC8223670 33355155

[72] J. S. Carter , S. Karczewski , D. D. DeCator , and A. M. Hollowell , “Ethnic Differences in Impact of Physical Activity Program on Psychological Symptoms in Youth,” Journal of Physical Activity & Health 14, no. 4 (2017): 283–289, 10.1123/jpah.2016-0450. 28032816

[73] M. K. Tennfjord , M. F. Strand , N. Østby , K. M. T. Harbø , and P. M. Fredriksen , “A School‐Based Physical Activity Intervention on Psychosocial Health Outcomes Among 11‐ and 12‐Year‐Olds—HOPP‐Project,” Scandinavian Journal of Medicine & Science in Sports 33, no. 4 (2023): 455–464, 10.1111/sms.14278. 36420609

[74] I. Kliziene , A. Emeljanovas , and M. Dubosas , “School Physical Education Program Impact on Psychological Well‐Being and Cognitive Ability of Primary School Children,” Teor Metod Fiz Vihov 23, no. 2 (2023): 290–298, 10.17309/tmfv.2023.2.19.

[75] M. Malakellis , E. Hoare , A. Sanigorski , et al., “School‐Based Systems Change for Obesity Prevention in Adolescents: Outcomes of the Australian Capital Territory ‘It's Your Move!’,” Australian and New Zealand Journal of Public Health 41, no. 5 (2017): 490–496, 10.1111/1753-6405.12696. 28749562

[76] E. A. Baker , M. Elliott , E. Barnidge , et al., “Implementing and Evaluating Environmental and Policy Interventions for Promoting Physical Activity in Rural Schools,” Journal of School Health 87, no. 7 (2017): 538–545, 10.1111/josh.12522. 28580669

[77] N. L. Holt , Z. L. Sehn , J. C. Spence , A. S. Newton , and G. D. C. Ball , “Physical Education and Sport Programs at an Inner City School: Exploring Possibilities for Positive Youth Development,” Physical Education and Sport Pedagogy 17, no. 1 (2012): 97–113, 10.1080/17408989.2010.548062.

[78] H. E. Mills , M. A. McNarry , G. Stratton , S. D. Mellalieu , and K. A. Mackintosh , “Investigating the Effectiveness on Educational Attainment and Behaviour of Commando Joe's: A School‐Based, Military‐Ethos Intervention,” Archives of Exercise in Health and Disease 5, no. 1/2 (2015): 377–384, 10.5628/aehd.v5i1-2.185.

[79] H. Diao , H. Wang , L. Yang , and T. Li , “The Impacts of Multiple Obesity‐Related Interventions on Quality of Life in Children and Adolescents: A Randomized Controlled Trial,” Health and Quality of Life Outcomes 18, no. 1 (2020): 213, 10.1186/s12955-020-01459-0. 32631401 PMC7336614

[80] A. Hall , L. Wolfenden , A. Shoesmith , et al., “The Impact of an Implementation Intervention That Increased School's Delivery of a Mandatory Physical Activity Policy on Student Outcomes: A Cluster‐Randomised Controlled Trial,” Journal of Science and Medicine in Sport 25, no. 4 (2022): 321–326, 10.1016/j.jsams.2021.12.005. 35074278

[81] A. Masini , S. Marini , A. Ceciliani , et al., “The Effects of an Active Breaks Intervention on Physical and Cognitive Performance: Results From the I‐MOVE Study,” Journal of Public Health 45, no. 4 (2023): 919–929, 10.1093/pubmed/fdad102. 37403403

[82] U. Meyer , C. Schindler , L. Zahner , et al., “Long‐Term Effect of a School‐Based Physical Activity Program (KISS) on Fitness and Adiposity in Children: A Cluster‐Randomized Controlled Trial,” PLoS ONE 9, no. 2 (2014): e87929, 10.1371/journal.pone.0087929. 24498404 PMC3912178

[83] L. B. Azevedo , D. Burges Watson , C. Haighton , and J. Adams , “The Effect of Dance Mat Exergaming Systems on Physical Activity and Health—Related Outcomes in Secondary Schools: Results From a Natural Experiment,” BMC Public Health 14, no. 1 (2014): 951, 10.1186/1471-2458-14-951. 25217144 PMC4169828

[84] M. Hamer , D. Aggio , G. Knock , C. Kipps , A. Shankar , and L. Smith , “Effect of Major School Playground Reconstruction on Physical Activity and Sedentary Behaviour: Camden Active Spaces,” BMC Public Health 17, no. 1 (2017): 552, 10.1186/s12889-017-4483-5. 28592241 PMC5463303

[85] S. E. Kvalø and I. K. Natlandsmyr , “The Effect of Physical‐Activity Intervention on Children's Health‐Related Quality of Life,” Scandinavian Journal of Public Health 49, no. 5 (2021): 539–545, 10.1177/1403494820971493. 33228472

[86] M. Rocher , B. Silva , G. Cruz , R. Bentes , J. Lloret , and E. Inglés , “Benefits of Outdoor Sports in Blue Spaces. The Case of School Nautical Activities in Viana do Castelo,” International Journal of Environmental Research and Public Health 17, no. 22 (2020): 8470, 10.3390/ijerph17228470. 33207658 PMC7697647

[87] A. d. S. Bandeira , M. W. Beets , P. M. da Silveira , et al., “Efforts on Changing Lifestyle Behaviors May Not be Enough to Improve Health‐Related Quality of Life Among Adolescents: A Cluster‐Randomized Controlled Trial,” Frontiers in Psychology 12 (2021): 614628, 10.3389/fpsyg.2021.614628. 33679529 PMC7929984

[88] L. S. Elinder , N. Heinemans , J. Hagberg , A. K. Quetel , and M. Hagströmer , “A Participatory and Capacity‐Building Approach to Healthy Eating and Physical Activity—SCIP‐School: a 2‐Year Controlled Trial,” International Journal of Behavioral Nutrition and Physical Activity 9, no. 1 (2012): 145, 10.1186/1479-5868-9-145. 23245473 PMC3545832

[89] D. R. Lubans , P. J. Morgan , A. D. Okely , et al., “Preventing Obesity Among Adolescent Girls: One‐Year Outcomes of the Nutrition and Enjoyable Activity for Teen Girls (NEAT Girls) Cluster Randomized Controlled Trial,” Archives of Pediatrics & Adolescent Medicine 166, no. 9 (2012): 821–827, 10.1001/archpediatrics.2012.41. 22566517

[90] Z. Zhou , Y. Zhou , F. V. Ferraro , A. Hooton , and C. Ribchester , “The Effects of Latino Dance Intervention on Academic and General Self‐Efficacy With Left‐Behind Children: An Experimental Study in China,” Frontiers in Psychology 14 (2023): 1107233, 10.3389/fpsyg.2023.1107233. 37205070 PMC10187033

[91] K. Ryom , J. M. Wikman , and R. Stelter , “Supporting Self‐Concept in School Settings Targeting Migrant Background Boys,” Scandinavian Journal of Educational Research 65, no. 4 (2021): 676–692, 10.1080/00313831.2020.1739136.

[92] R. L. Woodgate and C. M. Sigurdson , “Building School‐Based Cardiovascular Health Promotion Capacity in Youth: A Mixed Methods Study,” BMC Public Health 15, no. 1 (2015): 421, 10.1186/s12889-015-1759-5. 25909502 PMC4416265

[93] V. Villarreal and J. E. Gonzalez , “Extracurricular Activity Participation of Hispanic Students: Implications for Social Capital Outcomes,” International Journal of School and Educational Psychology 4, no. 3 (2016): 201–212, 10.1080/21683603.2015.1119092.

[94] A. Spruit , M. Assink , E. Van Vugt , C. Van Der Put , and G. J. Stams , “The Effects of Physical Activity Interventions on Psychosocial Outcomes in Adolescents: A Meta‐Analytic Review,” Clinical Psychology Review 45 (2016): 56–71, 10.1016/j.cpr.2016.03.006. 27064552

[95] L. Asfour , M. Koussa , T. Perrino , M. Stoutenberg , and G. Prado , “The Association of Organized and Unorganized Physical Activity and Sedentary Behavior With Internalizing and Externalizing Symptoms in Hispanic Adolescents,” Child and Adolescent Mental Health 21, no. 2 (2016): 109–114, 10.1111/camh.12139. 27346986 PMC4915383

[96] F. Alawie , E. Olivier , and V. Dupéré , “Can Physical Activity Protect Young Adolescents With Difficult Temperaments and Exposed to Family Adversity From Internalizing and Externalizing Problems? Yes, but…,” Journal of Early Adolescence 45, no. 1 (2024): 15–43, 10.1177/02724316231224812.

[97] M. W. Beets , R. G. Weaver , J. P. A. Ioannidis , et al., “Identification and Evaluation of Risk of Generalizability Biases in Pilot Versus Efficacy/Effectiveness Trials: A Systematic Review and Meta‐Analysis,” International Journal of Behavioral Nutrition and Physical Activity 17, no. 1 (2020): 19, 10.1186/s12966-020-0918-y. 32046735 PMC7014944

[98] K. Opstoel , L. Chapelle , F. J. Prins , et al., “Personal and Social Development in Physical Education and Sports: A Review Study,” European Physical Education Review 26, no. 4 (2020): 797–813, 10.1177/1356336X19882054.

[99] G. Arslan, PhD , “School Belongingness, Well‐Being, and Mental Health Among Adolescents: Exploring the Role of Loneliness,” Australian Journal of Psychology 73, no. 1 (2021): 70–80, 10.1080/00049530.2021.1904499.

[100] E. V. Bania , C. Eckhoff , and S. Kvernmo , “Not Engaged in Education, Employment or Training (NEET) in an Arctic Sociocultural Context: the NAAHS Cohort Study,” BMJ Open 9, no. 3 (2019): e023705, 10.1136/bmjopen-2018-023705. PMC6475364 30904841

[101] N. Leigh‐Hunt , D. Bagguley , K. Bash , et al., “An Overview of Systematic Reviews on the Public Health Consequences of Social Isolation and Loneliness,” Public Health 152 (2017): 157–171, 10.1016/j.puhe.2017.07.035. 28915435

[102] A. L. Jakobsen , C. D. Hansen , and J. H. Andersen , “The Association Between Perceived Social Support in Adolescence and Positive Mental Health Outcomes in Early Adulthood: A Prospective Cohort Study,” Scandinavian Journal of Public Health 50, no. 3 (2022): 404–411, 10.1177/1403494821993718. 33645305

[103] J. C. Bikomeye , J. Balza , and K. M. Beyer , “The Impact of Schoolyard Greening on Children's Physical Activity and Socioemotional Health: A Systematic Review of Experimental Studies,” International Journal of Environmental Research and Public Health 18, no. 2 (2021): 535, 10.3390/ijerph18020535. 33561082 PMC7827958

[104] A. C. M. Suga , A. A. d. P. da Silva , J. R. Brey , P. H. Guerra , and C. R. Rodriguez‐Añez , “Effects of Interventions for Promoting Physical Activity During Recess in Elementary Schools: A Systematic Review,” Jornal de Pediatria 97 (2021): 585–594, 10.1016/j.jped.2021.02.005. 33773960 PMC9432283

[105] S. M. Dyrstad , S. E. Kvalø , M. Alstveit , and I. Skage , “Physically Active Academic Lessons: Acceptance, Barriers and Facilitators for Implementation,” BMC Public Health 18, no. 1 (2018): 322, 10.1186/s12889-018-5205-3. 29510699 PMC5839008

[106] L. Martyn , H. Bigelow , J. D. Graham , M. Ogrodnik , D. Chiodo , and B. Fenesi , “A Mixed Method Investigation of Teacher‐Identified Barriers, Facilitators and Recommendations to Implementing Daily Physical Activity in Ontario Elementary Schools,” BMC Public Health 22, no. 1 (2022): 1986, 10.1186/s12889-022-14359-3. 36316654 PMC9619006

[107] E. Norris , T. van Steen , A. Direito , and E. Stamatakis , “Physically Active Lessons in Schools and Their Impact on Physical Activity, Educational, Health and Cognition Outcomes: A Systematic Review and Meta‐Analysis,” British Journal of Sports Medicine 54, no. 14 (2020): 826–838, 10.1136/bjsports-2018-100502. 31619381

[108] A. Daly‐Smith , T. Quarmby , V. S. J. Archbold , et al., “Using a Multi‐Stakeholder Experience‐Based Design Process to Co‐Develop the Creating Active Schools Framework,” International Journal of Behavioral Nutrition and Physical Activity 17, no. 1 (2020): 13, 10.1186/s12966-020-0917-z. 32028968 PMC7006100

[109] R. L. Pearl , T. A. Wadden , and J. M. Jakicic , “Is Weight Stigma Associated With Physical Activity? A Systematic Review,” Obesity 29, no. 12 (2021): 1994–2012, 10.1002/oby.23274. 34747131 PMC8612947

[110] O. B. Rakaa , M. Bassiri , and S. Lotfi , “Adapted Pedagogical Strategies in Inclusive Physical Education for Students With Special Educational Needs: A Systematic Review,” Pedagogy of Physical Culture and Sports 29, no. 2 (2025): 67–85, 10.15561/26649837.2025.0201.

