# Supplementary material for: The Effectiveness of School‐Based Physical Activity Promotion on Mental Health Among Children and Adolescents: A Systematic Review
Source: Scand J Med Sci Sports. 2025 Oct 19;35(10):e70150. doi: 10.1111/sms.70150 (PMC12536061; doi:10.1111/sms.70150)
Supplement: Supplementary file 1 — Appendix S1: sms70150‐sup‐0001‐AppendixS1.docx. [file SMS-35-e70150-s002.docx]

Supporting Information S1

**Amendments to information provided in the protocol**

The protocol (PROSPERO registration number CRD42022355274) can be found in <https://www.crd.york.ac.uk/PROSPERO/view/CRD42022355274>

1. **Amendment:** The title of the review has been changed.
   **Reason:** The title was revised to better reflect the content of the manuscript.
   **The stage of the review process:** The title was revised during the finalization of the manuscript.
2. **Amendment:** The review objectives were expanded to also examine which types of physical activity promotion are most effective in supporting mental health.
   **Reason:** While reviewing the background literature, it became evident that the type of intervention and the implementer play a significant role in school-based settings.
   **The stage of the review process:** The decision to expand the review objectives was made after reading the full texts, but prior to data extraction, upon recognizing that the included studies encompassed a wide variety of intervention types, thereby allowing for comparative analysis.
3. **Amendment:** The database searches were limited to English-language publications, although the original protocol had planned to include articles published in Finnish as well.
   **Reason:** Not all members of the final review team are Finnish speakers.
   **The stage of the review process:** The decision was made during the development of the search strategy.
4. **Amendment:** The publication date range of the database searches was extended up to March 28, 2024, during the updated search. The date of the updated search was March 28, 2024, as the original search had been conducted on June 27, 2022.
   **Reason:** As the original search results were nearly two years old, an updated search was conducted to ensure the inclusion of the most recent studies in the review.
   **The stage of the review process:** After the first version of the manuscript had already been completed.
5. **Amendment:** The inclusion criteria were refined to also incorporate mixed methods studies, in addition to quantitative and qualitative research.
   **Reason:** Mixed methods studies were included in the review from the outset, but the wording of the inclusion criteria was refined to improve clarity.
   **The stage of the review process:** During the manuscript writing phase.
6. **Amendment:** The exclusion criteria were refined to specify the exclusion of case studies with fewer than five participants, and were expanded to encompass conference or symposium proceedings and theses.
   **Reason:** It was observed that peer review status was often unclear for conference proceedings and theses. The term "case study" is used very broadly in the literature, but the aim was to exclude only studies with a very small sample size.
   **The stage of the review process:** After conducting test searches in the databases and reviewing their results.
7. **Amendment:** The Mixed Methods Appraisal Tool (MMAT) was chosen for the quality assessment.
   **Reason:** Due to the diverse designs of the included studies, a quality appraisal tool applicable to all study designs was chosen to ensure consistency in the assessment.
   **The stage of the review process:** Before initiating the quality assessment, once the included studies had already been selected.
8. **Amendment:** Doctoral researcher Mervi Haavanlammi was removed from the review team, and doctoral researcher Terhi Koivumäki was added to the team.
   **Reason:** MH withdrew from the project due to personal reasons, and TK joined the team as her replacement.
   **The stage of the review process:** After the database searches had been completed, but before the screening of titles and abstracts.
9. **Amendment:** Professor Alberto Ruiz-Ariza was added as a member of the review team.
   **Reason:** ARA made significant contributions to the design of the study and the search strategy, as well as to the writing of the manuscript.
   **The stage of the review process:** During the manuscript writing phase.
10. **Amendment:** The end date of the review timeline was extended until June 20, 2024.
    **Reason:** An updated search was decided to be conducted for the review, which made it necessary to extend the review timeline.
    **The stage of the review process:** Following the completion of the first draft of the manuscript.
