# Supplementary material for: The Effectiveness of School‐Based Physical Activity Promotion on Mental Health Among Children and Adolescents: A Systematic Review
Source: Scand J Med Sci Sports. 2025 Oct 19;35(10):e70150. doi: 10.1111/sms.70150 (PMC12536061; doi:10.1111/sms.70150)
Supplement: Supplementary file 2 — Appendix S2: sms70150‐sup‐0002‐AppendixS2.docx. [file SMS-35-e70150-s001.docx]

| Supporting Information S2  Table S1. Effect of the school-based physical activity promotion on internalizing symptoms in quantitative studies. | | | | | | | | | | | | |
| --- | --- | --- | --- | --- | --- | --- | --- | --- | --- | --- | --- | --- |
| Authors (year) | | Intervention | | | | |  | Control | | | | Outcome / Instrument |
|  | | n | | Mean (SD) | | |  | n | | Mean (SD) | |  |
| Barnes et al. (2021)  Baseline  9 months | | 485 478 | | NR 69.56 (19.15) | | |  | 330 323 | | NR 72.13 (19.98) | | Emotional functioning / Pediatric Quality of Life Inventory |
| Bunketorp Käll et al. (2015)  Baseline  Follow-up  0-3-Graders  4-6-Graders | | 85 97 | | Not assessed  1.0 (1.2) 1.9 (1.7) | | |  | 95 72 | | Not assessed  1.3 (1.6) 2.1 (2.3) | | Emotional symptoms /  Strengths and Difficulties Questionnaire |
| Carter et al. (2017)  Baseline  1 year | | 78 NR | | NR NR | | |  | 33 NR | | NR NR | | Internalizing symptoms /  Child Behavior Checklist and  Teacher Report Form |
| Grillich et al. (2016)  Baseline  1.5 year | | NR NR | | NR NR | | |  | NR NR | | NR NR | | Moods and emotions /  KIDSCREEN-52 |
| Kliziene et al. (2023)  Pre-test / Somatic anxiety  Boys  Girls  Post-test / Somatic anxiety  Boys  Girls  Pre-test / Personality anxiety  Boys  Girls  Post-test / Personality anxiety  Boys  Girls  Pre-test / Social anxiety  Boys  Girls  Post-test / Social anxiety   Boys  Girls | | 102 100  102 100  102 100  102 100  102 100  102 100 | | 4.97 (1.05) 4.98 (1.13)  4.63 (1.00) 4.92 (1.00)  3.49 (1.15) 3.71 (0.85)  2.68 (0.98) 3.41 (0.55)  6.28 (1.08) 6.03 (1.32)  4.26 (1.41) 5.30 (1.25) | | |  |  | |  | | Anxiety /  Revised Children's Manifest Anxiety Scale |
| Lubans et al. (2021)  Baseline / Internalizing problems  1 year / Internalizing problems  Baseline / Perceived stress  1 year / Perceived stress | | 337 214 337 214 | | 5.4 (3.2) 5.3 (3.1) 19.1 (6.1) 19.5 (6.4) | | |  | 333 220 333 220 | | 5.5 (3.2) 5.6 (3.3)  18.8 (6.2) 19.6 (6.2) | | Internalizing problems /  Strengths and Difficulties Questionnaire Perceived stress /  Perceived Stress Scale |
| Malakellis et al. (2017)  Baseline  2 years | | 628 499 | | NR NR | | |  | 252 157 | | NR NR | | Depressive symptoms /  The Adolescent Behaviours, Attitudes and Knowledge Questionnaire |
| Nathan et al. (2013)  Baseline  Follow-up | | 63 | | Not assessed 3.27 (2.61) | | |  | 79 | | Not assessed 3.32 (2.07) | | Emotional symptoms /  Strengths and Difficulties Questionnaire |
| Tennfjord et al. (2023)  Baseline  2016 follow-up  2017 follow-up  2018 follow-up  2019 follow-up | | NR 387 377 286 267 | | Not assessed 3.82 (2.56) 4.15 (2.52) 3.99 (2.54) 4.30 (2.61) | | |  | NR 213 175 162 160 | | Not assessed 3.05 (2.63) 3.45 (2.51) 3.48 (2.55) 3.68 (2.66) | | Internalizing difficulties /  Strengths and Difficulties Questionnaire |
| van Dijk-Wesselius et al. (2018)  Baseline  1 year  2 years | | 351 360 331 | | 3.77 (0.81) 3.58 (0.82) 3.59 (0.75) | | |  | 355 322 312 | | 3.75 (0.82) 3.64 (0.73) 3.59 (0.75) | | Emotional functioning /  Pediatric Quality of Life Inventory |
| n = sample size; SD = standard deviation; NR = not reported | | | | | | | | | | | | |
| Table S2. Effect of the school-based physical activity promotion on externalizing symptoms in quantitative studies. | | | | | | | | | | | | |
| Authors (year) | Intervention | | | |  | Control | | | | | Outcome / Instrument | |
|  | n | | Mean (SD) | |  | n | | | Mean (SD) | |  | |
| Bunketorp Käll et al. (2015)  Baseline  Follow-up  0-3-Graders / Conduct problems  0-3-Graders / Hyperactivity  4-6-Graders / Conduct problems  4-6-Graders / Hyperactivity | 85 85 97 97 | | Not assessed  NR 2.1 (2.0) 1.4 (1.2) 2.9 (2.0) | |  | 95 95 72 72 | | | Not assessed  1.0 (1.1) 2.6 (2.5) 1.7 (1.3) 3.4 (2.0) | | Conduct problems and  hyperactivity / Strengths and Difficulties Questionnaire | |
| Carter et al. (2017)  Baseline  1 year | 78 NR | | NR NR | |  | 33 NR | | | NR NR | | Externalizing symptoms /  Child Behavior Checklist and  Teacher Report Form | |
| Mills et al. (2015)  Baseline   1 year | 249 NR | | NR NR | |  | 146 NR | | | NR NR | | Problem behavior / Nisonger Child Behavior Rating | |
| Nathan et al. (2013)  Baseline  Follow-up | 63 | | Not assessed 2.88 (2.09) | |  | 79 | | | Not assessed 2.96 (1.66) | | Hyperactivity /  Strengths and Difficulties Questionnaire | |
| Tennfjord et al. (2023)  Baseline  2016 follow-up  2017 follow-up  2018 follow-up  2019 follow-up | NR 387 377 286 267 | | Not assessed 4.93 (2.75) 5.13 (2.52) 4.89 (2.54) 5.18 (2.61) | |  | NR 213 175 162 160 | | | Not assessed 4.24 (2.77) 4.42 (2.65) 4.43 (2.55) 4.30 (2.66) | | Externalizing difficulties /  Strengths and Difficulties Questionnaire | |
| n = sample size; SD = standard deviation; NR = not reported | | | | | | | | | | | | |

| Table S3. Effect of the school-based physical activity promotion on mental well-being in quantitative studies. | | | | | | |
| --- | --- | --- | --- | --- | --- | --- |
| Authors (year) | Intervention | |  | Control | | Outcome / Instrument |
|  | n | Mean (SD) |  | n | Mean (SD) |  |
| Allender et al. (2021)  Baseline  2-year follow-up  4-year follow-up | 565 677 578 | NR NR NR |  | 530 903 821 | NR NR NR | Psychosocial HRQoL /  Pediatric Quality of Life Inventory |
| Azevedo et al. (2014)  Baseline  1 year | 226 148 | 50.9 (10.9) 50.4 (10.7) |  | 179 131 | 52.9 (9.1) 48.8 (9.3) | Psychological well-being /  KIDSCREEN-27 |
| Bandeira et al. (2021)  Baseline  9 months | 538 434 | 45.8 (10.8) 43.5 (NR) |  | 383 300 | 45.6 (12.5) 45.0 (NR) | Psychological well-being / KIDSCREEN-27 |
| Bunketorp Käll et al. (2015)  Baseline  Follow-up  0-3-Graders  4-6-Graders | 85 97 | Not assessed  29.9 (2.8) 30.8 (3.8) |  | 95 72 | Not assessed  29.8 (3.0) 30.0 (3.5) | Psychological well-being /  KIDSCREEN-27 |
| Christiansen et al. (2018)  Baseline  9 months | 1301 1301 | 3.28 (0.68) 3.36 (0.67) |  | 1496 1496 | 3.28 (0.68) 3.35 (0.69) | Global self-worth /  Self-Perception Profile for Children |
| Christiansen et al. (2022)  Baseline  9 months | 1541 NR | NR NR |  | 1343 NR | NR NR | Psychological well-being / KIDSCREEN-27 |
| Diao et al. (2020)  Baseline  1 year | 547 518 | 38.66 (7.25 41.44 (7.33) |  | 445 430 | 40.12 (6.81) 40.90 (7.25) | Psychological QoL /  The Adolescent Quality of Life Scale |
| Elinder et al. (2012)  Baseline  2 years | 471 405 | NR NR |  | 328 282 | NR NR | Self-esteem /  KEY to School Health Questionnaire |
| Grillich et al. (2016)  Baseline / Psychological well-being  1.5 years / Psychological well-being  Baseline / Sense of coherence  1.5 years / Sense of coherence | NR NR NR NR | NR NR NR NR |  | NR NR NR NR | NR NR NR NR | Psychological well-being /  KIDSCREEN-52 Sense of coherence / Bettge, S. (2004) |
| Hall et al. (2022)  Baseline  1 year | 657 | Not assessed 84.01 (12.79) |  | 563 | Not assessed 83.81 (13.44) | Psychosocial HRQoL / Pediatric Quality of Life Inventory |
| Harrington et al. (2018) & (2019)  Baseline   14 months | 700 730 | Median (IQR) 3.5 (2.2-4.2) 3.3 (2.7-4.0) |  | 760 535 | Median (IQR) 3.5 (2.8-4.2) 3.3 (2.8-4.0) | Self-esteem / Physical Self-perception Profile Questionnaire |
| Kvalo & Natlandsmyr (2021)  Baseline  11 months | 161 208 | 55.00 (9.6) 56.52 (NR) |  | 171 197 | 53.5 (9.2) 53.50 (NR) | Psychological well-being /  KIDSCREEN-27 |
| Lubans et al. (2012)  Baseline  1 year | 178  141 | 4.16 (1.09) 4.09 (1.10) |  | 179 153 | 4.28 (1.01) 4.29 (0.99) | Global self-esteem /  Marsh's Physical Self-description Questionnaire |
| Lubans et al. (2021)  Baseline   1 year | 337 214 | 24.9 (4.8) 25.0 (4.5) |  | 333 220 | 23.9 (5.0) 24.6 (4.9) | Mental well-being /  Warwick-Edinburgh Mental Well-being Scale |
| Masini et al. (2023)  Baseline  1.5 years | 83 83 | 72.06 (15.04) 78.50 (11.88) |  | 50 50 | 67.35 (15.87) 74.94 (12.43) | Psychosocial HRQoL /  Pediatric Quality of Life Inventory |
| Meyer et al. (2014)  Baseline   3 years | 136 136 | 53.3 (6.8) 53.0 (6.8) |  | 55 55 | 53.4 (6.3) 51.7 (9.6) | Psychological QoL / The Child Health Questionnaire |
| Nathan et al. (2013)  Baseline  Follow-up | 63 | Not assessed 3.59 (1.07) |  | 79 | Not assessed 3.69 (1.06) | Resilience / The Connor-Davidson Resilience Scale |
| Rocher et al. (2020)  Cross-sectional data | 595 | Not reported |  |  |  | Mental health and well-being |
| Ryom et al. (2021)  Baseline  2 years | NR 35 | NR NR |  | NR 26 | NR NR | General self-concept /  The Self-Description Questionnaire II |
| Woodgate & Sigurdson (2015)  Pre-test / Caring  Post-test / Caring  Pre-test / Character  Post-test / Character  Pre-test / Competence  Post-test / Competence  Pre-test / Connection  Post-test / Connection  Pre-test / Confidence  Post-test / Confidence | 20 20 20 20 20 20 20 20 20 20 | 69.99 (16.67) 71.49 (18.55) 77.30 (15.96)  75.33 (9.70) 69.73 (16.24) 69.27 (9.87) 78.24 (11.00) 77.67 (9.94) 82.58 (12.87) 74.69 (12.79) |  |  |  | Positive Youth Development / PYD.2 |
| Zhou et al. (2023)  Pre-test / General self-efficacy  Post-test / General self-efficacy  Pre-test / Self-esteem  Post-test / Self-esteem | 305 305 305 305 | 17.90 (4.83) 25.55 (6.08) 15.59 (2.93) 29.19 (4.48) |  |  |  | General self-efficacy / Self-efficacy Scale by Schwarzer & Jerusalem (1995) Self-esteem /  Rosenberg Self-esteem Scale |
| n = sample size; SD = standard deviation; NR = not reported; HRQoL = health related quality of life; QoL = quality of life | | | | | | |

| Table S4. Effect of the school-based physical activity promotion on social well-being in quantitative studies. | | | | | | |
| --- | --- | --- | --- | --- | --- | --- |
| Authors (year) | Intervention | |  | Control | | Outcome / Instrument |
|  | n | Mean (SD) |  | n | Mean (SD) |  |
| Barnes et al. (2021)  Baseline  9 months | 485 478 | NR 82.57 (17.13) |  | 330 323 | NR 83.41 (17.94) | Social functioning /  Pediatric Quality of Life Inventory |
| Bunketorp Käll et al. (2015)  Baseline  Follow-up  0-3-Graders / Prosocial behavior  0-3-Graders / Peer problems  4-6-Graders / Prosocial behavior  4-6-Graders / Peer problems | 85 85 97 97 | Not assessed  1.2 (1.5) 0.8 (1.1) 1.7 (1.5) 1.5 (1.6) |  | 95 95 72 72 | Not assessed  1.2 (1.4) 1.0 (1.3) 2.0 (1.7) 1.6 (1.8) | Prosocial behavior and peer problems / Strengths and Difficulties Questionnaire |
| Christiansen et al. (2018)  Baseline  9 months | 1301 1301 | 2.94 (0.77) 3.03 (0.78) |  | 1496 1496 | 2.92 (0.76) 2.99 (0.78) | Social competence /  Self-Perception Profile for Children |
| Diao et al. (2020)  Baseline  1 year | 547 518 | 50.99 (8.19) 53.19 (8.88) |  | 445 430 | 50.77 (8.41) 52.53 (8.76) | Social QoL /  The Adolescent Quality of Life Scale |
| Mills et al. (2015)  Baseline  1 year | 249 NR | NR NR |  | 146 NR | NR NR | Positive social behavior /  Nisonger Child Behavior Rating |
| Nathan et al. (2013)  Baseline / Prosocial behavior  Follow-up / Prosocial behavior  Baseline / Peer problems  Follow-up / Peer problems | 63 63 | Not assessed 8.56 (1.44) Not assessed 2.95 (2.05) |  | 79 79 | Not assessed 8.05 (2.21) Not assessed 3.19 (1.57) | Prosocial behavior /  Strengths and Difficulties Questionnaire Peer problems /  Strengths and Difficulties Questionnaire |
| Rocher et al. (2020)  Cross-sectional data | 595 | Not reported |  |  |  | Social behavior and Sense of Community |
| Ryom et al. (2021)  Baseline / Relations to peers  2 years / Relations to peers  Baseline / Social relations  2 years / Social relations | NR 35 NR 35 | NR NR NR NR |  | NR 26 NR 26 | NR NR NR NR | Relations to peers / The Self-Description Questionnaire II Social relations / The Youth Sport Environment Questionnaire (modified to class environment) |
| van Dijk-Wesselius et al. (2018)  Baseline / Prosocial behavior  1 year / Prosocial behavior  2 years / Prosocial behavior  Baseline / Peer problems  1 year / Peer problems  2 years / Peer problems  Baseline / Social support  1 year / Social support  2 years / Social support | 351 360 331 351 360 331 351 360 331 | 2.24 (0.60) 2.26 (0.61) 2.23 (0.57) 0.63 (0.72) 0.46 (0.63) 0.49 (0.68) 1.85 (0.96) 2.18 (0.65) 2.27 (0.54) |  | 355 322 312 355 322 312 355 322 312 | 2.23 (0.54) 2.23 (0.56) 2.33 (0.49) 0.50 (0.66) 0.46 (0.62) 0.37 (0.57) 2.30 (0.61) 2.19 (0.58) 2.29 (0.51) | Prosocial behavior /  Strengths and Difficulties Questionnaire Peer problems /  Strengths and Difficulties Questionnaire Social support /  Dutch instrument for assessing school children's social functioning |
| Villarreal & Gonzalez (2016)  Cross-sectional data /   Prosocial behavior  Cross-sectional data /   Prosocial peer affiliation | 186  186 | Not reported  Not reported |  | 186  186 | Not reported  Not reported | Prosocial behavior /  Strengths and Difficulties Questionnaire Prosocial peer affiliation /  Adapted survey of Mahoney & Stattin (2000) |
| n = sample size; SD = standard deviation; NR = not reported; QoL = quality of life | | | | | | |

| Table S5. The quality of the evidence regarding mental health outcomes. | | | | | |
| --- | --- | --- | --- | --- | --- |
| Synthesis of effects | Type of data | Study designs | Informants | Age range | Quality of studies |
| Internalizing symptoms (12 studies) | The evidence is based on  both quantitative and qualitative data, with a stronger emphasis on quantitative research. | The evidence is based on  various study designs. | The results are mostly  based on self-reported data but also include some studies where the informant was a parent or a teacher. | The entire age range  covered by this review  (7-16 years) is well represented in the results. | Of the studies, 58% were  of good quality, containing at most one quality concern, while 42% of the studies exhibited multiple (≥2) quality concerns. |
| Externalizing symptoms (7 studies) | The evidence is primarily  based on quantitative data, supplemented by two qualitative studies. | The evidence is primarily  based on quasi-experimental studies, supplemented by two qualitative studies. | The results are based on  self-, parent- and teacher-reported data. | The findings mainly  concern children over the age of 10. | Of the studies, 43% were  of good quality (with ≤1 quality concern), while 57% of the studies exhibited multiple (≥2) quality concerns. |
| Mental well-being (23 studies) | The evidence is based on  both quantitative and qualitative data, with a stronger emphasis on quantitative research. | The evidence is based on  various study designs. | The included studies  primarily reported self-reported data, although a few also incorporated assessments from parents and teachers. | The entire age range  covered by this review  (7-16 years) is well represented in the results. | Of the studies, 52% were  of good quality (with ≤1 quality concern), while 48% of the studies exhibited multiple (≥2) quality concerns. |
| Social well-being (14 studies) | The evidence is based on  both quantitative and qualitative data. | The quantitative results are mostly based on quasi-experimental studies. | The results are based on  self-, parent- and teacher-reported data. | Although the entire age  range (7-16 years) covered by this review was represented in the studies, the majority focused on children and adolescents over the age of 10. | Of the studies, 57% were  of good quality (with ≤1 quality concern), while 43% of the studies exhibited multiple (≥2) quality concerns. |

| Table S6. The quality of the evidence regarding the type of PA promotion. | | | | | |
| --- | --- | --- | --- | --- | --- |
| Synthesis of effects | Type of data | Study designs | Informants | Age range | Quality of studies |
| Permanent environmental  modifications (5 studies) | The evidence is based on  both quantitative and qualitative data. | The evidence is based on  various study designs. | The results are based on  self-, parent- and teacher-reported data. | In all of the included studies, participants were under the age of 14. | Of the studies, 60% were  of good quality (with ≤1 quality concern), while 40% of the studies exhibited multiple (≥2) quality concerns. |
| PA promotion delivered  by an external actor outside  the school staff (7 studies) | The evidence is based on quantitative and mixed method studies. | The evidence is based on non-randomized study designs only. | The results are based on  self-, parent- and teacher-reported data. | The entire age range  covered by this review  (7-16 years) is well represented in the results. | Of the studies, only one (14%) was of good quality (with ≤1 quality concern), while other six studies (86%) exhibited multiple (≥2) quality concerns. |
| PA promotion delivered  by school staff following  a specific, predefined protocol (10 studies) | The evidence is based on  both quantitative and qualitative data, with a stronger emphasis on quantitative research. | The evidence is based on  various study designs. | The results are mostly  based on self-reported data but also include some studies where the informant was a parent or a teacher. | The entire age range  covered by this review  (7-16 years) is well represented in the results. | Of the studies, 50% were  of good quality (with ≤1 quality concern), while 50% of the studies exhibited multiple (≥2) quality concerns. |
| PA promotion delivered  by school staff without specific protocol (9 studies) | The evidence is based on quantitative data only. | The evidence is based on cRCT and quasi-experimental study designs. | The results are almost entirely based on self-reported data, with only one study incorporating parent-reported assessments. | The findings mainly  concern children over the age of 10. | Of the studies, 56% were  of good quality (with ≤1 quality concern), while 44% of the studies exhibited multiple (≥2) quality concerns. |
| PA = physical activity; cRCT = cluster randomized controlled trial | | | | | |
